# The Response of *Acinetobacter baumannii* to Hydrogen Sulfide Reveals Two Independent Persulfide-Sensing Systems and a Connection to Biofilm Regulation

**DOI:** 10.1128/mBio.01254-20

**Published:** 2020-06-23

**Authors:** Brenna J. C. Walsh, Jiefei Wang, Katherine A. Edmonds, Lauren D. Palmer, Yixiang Zhang, Jonathan C. Trinidad, Eric P. Skaar, David P. Giedroc

**Affiliations:** a Department of Chemistry, Indiana University, Bloomington, Indiana, USA; b Department of Molecular and Cellular Biochemistry, Indiana University, Bloomington, Indiana, USA; c Department of Pathology, Microbiology and Immunology, and Vanderbilt Institute for Infection, Immunology and Inflammation, Vanderbilt University Medical Center, Nashville, Tennessee, USA; d Laboratory for Biological Mass Spectrometry, Indiana University, Bloomington, Indiana, USA; National Institute of Child Health and Human Development (NICHD)

**Keywords:** *Acinetobacter baumannii*, hydrogen sulfide, reactive sulfur species, protein *S-*sulfuration, persulfide, persulfidation

## Abstract

Acinetobacter baumannii is an opportunistic nosocomial pathogen that is the causative agent of several serious infections in humans, including pneumonia, sepsis, and wound and burn infections. A. baumannii is also capable of forming proteinaceous biofilms on both abiotic and epithelial cell surfaces. Here, we investigate the response of A. baumannii toward sodium sulfide (Na_2_S), known to be associated with some biofilms at oxic/anoxic interfaces. The addition of exogenous inorganic sulfide reveals that A. baumannii encodes two persulfide-sensing transcriptional regulators, a primary σ^54^-dependent transcriptional activator (FisR), and a secondary system controlled by the persulfide-sensing biofilm growth-associated repressor (BigR), which is only induced by sulfide in a *fisR* deletion strain. FisR activates an operon encoding a sulfide oxidation/detoxification system similar to that characterized previously in Staphylococcus aureus, while BigR regulates a secondary persulfide dioxygenase (PDO2) as part of *yeeE-yedE-pdo2* sulfur detoxification operon, found previously in *Serratia* spp. Global *S-*sulfuration (persulfidation) mapping of the soluble proteome reveals 513 persulfidation targets well beyond FisR-regulated genes and includes five transcriptional regulators, most notably the master biofilm regulator BfmR and a poorly characterized catabolite regulatory protein (Crp). Both BfmR and Crp are well known to impact biofilm formation in A. baumannii and other organisms, respectively, suggesting that persulfidation of these regulators may control their activities. The implications of these findings on bacterial sulfide homeostasis, persulfide signaling, and biofilm formation are discussed.

## Abstract (precis)

Although hydrogen sulfide (H_2_S) has long been known as a respiratory poison, recent reports in numerous bacterial pathogens reveal that H_2_S and more downstream oxidized forms of sulfur collectedly termed reactive sulfur species (RSS) function as antioxidants to combat host efforts to clear the infection. Here, we present a comprehensive analysis of the transcriptional and proteomic response of A. baumannii to exogenous sulfide as a model for how this important human pathogen manages sulfide/RSS homeostasis. We show that A. baumannii is unique in that it encodes two independent persulfide sensing and detoxification pathways that govern the speciation of bioactive sulfur in cells. The secondary persulfide sensor, BigR, impacts the expression of biofilm-associated genes; in addition, we identify two other transcriptional regulators known or projected to regulate biofilm formation, BfmR and Crp, as highly persulfidated in sulfide-exposed cells. These findings significantly strengthen the connection between sulfide homeostasis and biofilm formation in an important human pathogen.

## INTRODUCTION

Acinetobacter baumannii is an increasing multidrug-resistant, opportunistic Gram-negative pathogen that causes widespread morbidity and significant mortality, particularly in immunocompromised patients (1). It is intrinsically resistant to a wide range of chemical and environmental insults, including long-term desiccation, nutrient starvation, and reactive oxygen species (ROS). A. baumannii is characterized by the ability to form biofilms on both abiotic and biotic surfaces and to withstand exposure to high concentrations of antibiotics, particularly in biofilms (2, 3). These factors, coupled with an alarming increase in the incidence of nosocomial infections has led the World Health Organization to classify A. baumannii as a priority 1 (critical) pathogen (4). An enhanced understanding of microbial physiology relevant to human infections and virulence in A. baumannii is needed to develop new avenues for antimicrobial therapies.

Recent findings that build on prior work (5) suggest that the upregulation of H_2_S biogenesis, both in Escherichia coli or uropathogenic E. coli (UPEC) and in Staphylococcus aureus, may well be a clinically important adaptive response during infections (6–8). Bacterial strains that lack H_2_S-biogenesis enzymes are more readily cleared in infected macrophages and are less resistant to leukocyte-mediated killing and perhaps other early immune responses. The *mstA* gene (formerly *sseA*) encodes 3-mercaptopyruvate sulfurtransferase (3MST) and is reported as a source of intracellular H_2_S in E. coli (5, 8). The bacterial loads of mice infected with a Δ*mstA* E. coli strain are significantly lower than in mice infected with wild-type E. coli in a burn infection model (6). Further, a Δ*mstA* E. coli strain, when passaged against sublethal doses of antibiotics, gives rise to a suppressor mutation that results in upregulation of an operon encoding enzymes involved in carbohydrate metabolism and the immediately adjacent *pspE* gene, encoding a single-domain sulfurtransferase, which is capable of generating significant H_2_S via reduction of the enzyme-bound persulfide (8). Finally, low levels of H_2_S enhance the respiration, energy production, and survival of Mycobacterium tuberculosis in infected mice (9). These findings suggest that the maintenance of endogenous H_2_S reduces the efficacy of first-line antibiotics and cellular redox balance in infected animals, consistent with other studies in clinically isolated multidrug-resistant UPEC strains (10) and in S. aureus (11). These data support the proposal that H_2_S functions as an infection-relevant antioxidant, which may be attributed to more downstream oxidized reactive sulfur species (RSS) (12). The origin of RSS in bacteria is not firmly established and may well differ among organisms, but major sources likely include sulfide:quinone oxidoreductase (SQR) which catalyzes the two-electron oxidation of sulfide to sulfane (sulfur-bonded) sulfur and l-cysteine catabolism (13, 14). Other possibilities are heme-based mechanisms (15–17), single-cysteine peroxiredoxins (18), and other nonenzymatic processes (19).

These beneficial effects of H_2_S, coupled with other metabolic considerations, e.g., that H_2_S is a substrate for cysteine synthase (20), suggest that cellular H_2_S levels must be tightly regulated (21). The discovery and characterization of organic persulfide (RSS)-sensing transcriptional regulators, including CstR, SqrR/BigR, and FisR, that regulate the expression of enzymes that are known or projected to reduce the cellular load of H_2_S and organic RSS led us to propose the concept of bacterial sulfide or RSS homeostasis (22–26). RSS homeostasis suggests that H_2_S and downstream RSS concentrations are actively managed by distinct structural classes of RSS-sensing repressors so as to allow the cell access to bioavailable sulfur in the form of more oxidized sulfur species, while limiting the deleterious impacts of sulfide poisoning and persulfidation of the low-molecular-weight (LMW) thiol pool and the proteome (11, 27). RSS are also proposed to function as heme-based signaling molecules in mammalian systems, for which there is now evidence (15–17).

Here, we describe the adaptive response of A. baumannii to external sulfide stress in an effort to identify potential participants in this regulatory response that models those processes that may be triggered by fluctuations in endogenous sulfide/RSS inside the cell. Transcriptomic and label-free proteomic analysis reveal a complex regulatory response to sulfide, involving a primary (FisR) and a secondary (BigR) persulfide-sensing transcriptional regulator, each of which controls the expression of sulfide oxidation and transport enzymes, including two candidate persulfide dioxygenases (PDO1 and PDO2). Metabolite profiling of LMW thiols, sulfide, and RSS in these cultures before and after the addition of Na_2_S reveals that although cysteine levels rise, A. baumannii is relatively resistant to perturbation of the cell-associated RSS and H_2_S. A. baumannii encodes 3MST, the deletion of which specifically lowers LMW persulfide levels. Sulfide increases the transcription of genes encoding an alternative cytochrome *bd* oxidase *cydAB*, as shown previously in E. coli (28) and M. tuberculosis (9), which is refractory to inhibition by H_2_S (10, 28). In addition, the cellular abundance of key ROS defense enzymes, including catalase, superoxide dismutase, and the alkylhydroperoxidase AhpCF, also increases. Protein *S-*sulfuration (persulfidation) profiling in A. baumannii reveals that ∼13% of the proteome is persulfidated and identifies potential regulatory targets of H_2_S/RSS stress, including two highly persulfidated transcriptional regulators, BfmR and Crp, known or projected to function in biofilm regulation.

## RESULTS

### LMW thiols and persulfides in wild-type and Δ*MST* cells.

**FIG 1:**
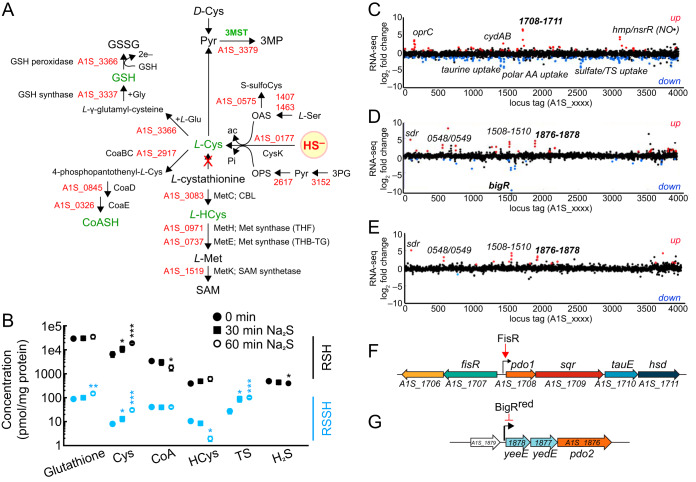
Profiling of LMW thiols and RSS and a transcriptomic analysis of A. baumannii in response to exogenous sulfide. (A) A. baumannii genes encoding proteins associated with the biosynthesis of LMW thiols and 3-mercaptopyruvate (3MP) from sulfide (HS^–^). OPS, *O*-phospho-l-serine; OAS, *O*-acetyl*-*l-serine; ac, acetate; pyr, pyruvate; CBL, cystathonine-β-lyase. (B) Endogenous concentrations of LMW thiols (RSH, black symbols), RSS (RSSH, TS, light blue symbols) and H_2_S (black) in WT A. baumannii and after the addition of 0.2 mM Na_2_S to mid-log-phase cultures. Statistical significance was established using a paired *t* test relative to the WT strain (*, *P ≤ *0.05; **, *P ≤ *0.01; ***, *P ≤ *0.001). (C to E) Fold change in transcription for WT+Na_2_S versus WT untreated (C), Δ*bigR* strain versus WT untreated (D), and Δ*bigR* strain versus Δ*bigR* mTn7(*bigR^C34A/C100A^*) strain (E), with both strains untreated. Genes with a significant fold change (*P* < 0.001) in three biological replicates are highlighted (red for a >2-fold increase or blue for a >2-fold decrease). (F and G) Genomic organization for A. baumannii FisR (F) and BigR (G) regulated operons derived from the data in panels C to E (see also Fig. 2). FisR, canonical σ^54^-transcriptional activator; PDO, persulfide dioxygenase; SQR, sulfide:quinone oxidoreductase; TauE, a putative sulfite exporter; *A1S_1711*, *hsd*, homoserine dehydrogenase; *A1S_1878*, *A1S_1877*, YeeE/YedE family transporter (COG2391) (37); BigR^red^, reduced BigR transcriptionally represses expression of its operon.

In previous studies in S. aureus and Enterococcus faecalis, we employed an isotope dilution, electrophile trapping method to estimate the concentrations of the major cellular thiols, thiol persulfides, and inorganic sulfide in cell lysates from mid-exponential-phase cells (11, 23, 29). In order to understand the regulatory response of A. baumannii ATCC 17978 to external sulfide stress, we used this approach to quantify endogenous levels of H_2_S, major cellular thiols and thiol persulfides in mid-log-phase wild-type (WT) cells and compare these levels to those in a strain lacking the 3MST (A1S_3379). 3MST is thought to be an important source of endogenously synthesized H_2_S from studies of E. coli (5, 6, 8). Note also that A. baumannii harbors complete glutathione (GSH) and coenzyme A (CoASH) biosynthetic pathways from cysteine, with homocysteine (HCys) derived from l-cystathionine (Fig. 1A).

We found that GSH is the major cellular thiol, followed by cysteine and CoASH, which are comparable in concentration to one another, with the concentration of homocysteine being about 10-fold lower than those of cysteine and CoASH and about equal to that of H_2_S (Fig. 1B). The addition of Na_2_S to WT cells results in a significant increase in cellular cysteine possibly as a result of increased flux through cysteine synthase (CysK) (Fig. 1A), with a corresponding increase in cysteine persulfide as well (Fig. 1B). A. baumannii harbors thiol persulfides for each of its major cellular thiols, with glutathione persulfide (GSSH) predominating (Fig. 1B). Exogenous Na_2_S perturbs these levels primarily by increasing GSSH, cysteine persulfide, and the inorganic species thiosulfate (S_2_O_3_^2–^ [TS]). Interestingly, the fraction of thiol persulfide is significantly lower for GSSH and cysteine persulfide, which represent ∼0.1% of their respective total thiol pools compared to that of CoASH persulfide which is ∼2% (Fig. 1B).

The impact of 3MST on RSS in cells was also investigated (see Fig. S1 in the supplemental material). Interestingly, deletion of 3MST (Δ*MST*) does not directly impact cellular H_2_S levels, but instead results in small decreases in GSH and HCys levels, with correspondingly larger (≥4-fold) decreases in endogenous GSSH, cysteine, and homocysteine persulfide levels (Fig. S1A and B). The addition of Na_2_S to Δ*MST* cells results in increases in the LMW thiol persulfide pools within 30 min, notably GSSH, which increases ∼8-fold to a level comparable to that of the WT strain (Fig. S1C). These data taken collectively reveal that 3MST significantly impacts endogenous sulfur speciation in cells and that some external Na_2_S is assimilated as TS and LMW thiol persulfides.

10.1128/mBio.01254-20.1 FIG S1 Profiling of LMW thiols and RSS in a Δ*MST* strain compared to the WT strain. (A and B) Endogenous levels of LMW thiols and H_2_S (A) and LMW persulfides and thiosulfate (TS) (B) in Δ*MST* strain compared to WT. (C) Change in RSS in Δ*MST* versus WT 30 min after the addition of Na_2_S to mid-log-phase cell cultures. The WT data shown here are also shown in Fig. 1 to facilitate comparisons. Statistical significance was established using a paired *t* test relative to the WT (***, *P ≤ *0.001,**, *P ≤ *0.01; *, *P ≤ *0.05). Download FIG S1, PDF file, 0.2 MB. Copyright © 2020 Walsh et al. 2020 Walsh et al. This content is distributed under the terms of the Creative Commons Attribution 4.0 International license.

### The σ^54^-transcriptional activator FisR controls the primary transcriptomic response to exogenous sulfide in *A. baumannii*.

**FIG 2:**
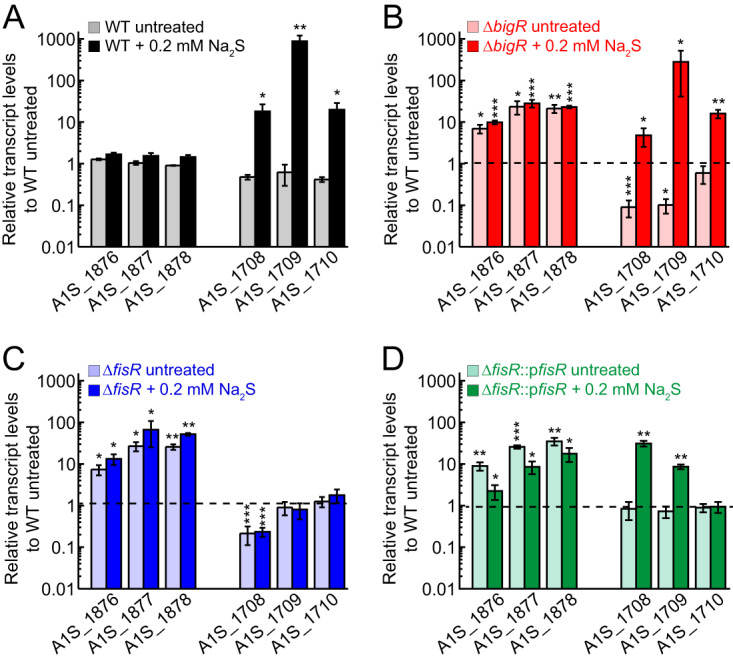
qRT-PCR analysis of A. baumannii and mutant strains. (A to D) Relative transcript levels of BigR (*A1S_1876-1878*) and FisR (*A1S_1708-1710*) regulated genes in WT (A), Δ*bigR* (B), Δ*fisR* (C), and Δ*fisR*::p*fisR* (D) strains with or without the addition of 0.2 mM Na_2_S. The horizontal dashed line in panels B to D represents untreated WT, as shown in panel A. Statistical significance was established using a paired *t* test relative to the WT strain (*, *P ≤ *0.05; **, *P ≤ *0.01; ***, *P ≤ *0.001).

To further investigate the impact of H_2_S on A. baumannii, we investigated the transcriptomic response of A. baumannii to the addition of the 0.2 mM Na_2_S. We initially compared the transcriptomic profiles of three strains: the WT strain, the Δ*bigR* strain, and a Δ*bigR* mTn7(*bigR^C34A/C100A^*) complemented strain. BigR (biofilm growth-associated repressor; A1S_1539) is an arsenic-repressor (ArsR)-superfamily transcriptional regulator expected to sense RSS on the basis of sequence similarity to other dithiol (C34, C100)-containing persulfide sensors characterized previously in Gram-negative bacteria, Rhodobacter capsulatus and Xylella fastidiosa, denoted SqrR and BigR, respectively (24, 25). These results are summarized in Fig. 1C to G and also Table S1A in the supplemental material, with key aspects of these findings confirmed by real-time quantitative reverse transcription PCR (qRT-PCR) (Fig. 2).

10.1128/mBio.01254-20.9 TABLE S1 (A) Transcriptionally up- or downregulated genes via RNA-seq analysis, related to Fig. 1. (B) Composite list of proteins identified in LC-MS/MS analysis of WT and WT+Na_2_S A. baumannii, related to Fig. 4 and to Fig. S4 and S5. (C) Composite list of proteins identified in LC-MS/MS analysis of Δ*fisR* and Δ*fisR*+Na_2_S A. baumannii strains, related to Fig. S4 and S6. (D) Composite list of proteins identified as *S*-sulfurated in WT A. baumannii, related to Fig. 5 and Fig. S7. Download Table S1, XLSX file, 0.3 MB. Copyright © 2020 Walsh et al. 2020 Walsh et al. This content is distributed under the terms of the Creative Commons Attribution 4.0 International license.

Disodium sulfide induces the expression of several operons. The most highly upregulated genes are *A1S_1708-1709-1710-1711*, which encode a persulfide dioxygenase (PDO1), a sulfide:quinone oxidoreductase (SQR), a putative TS/sulfite effluxer TauE, and a candidate homoserine dehydrogenase (Fig. 1C and F and Fig. 2A; see also Table S1A in the supplemental material). The first three genes correspond to those found in the RSS-regulated operon, *cst*, in S. aureus controlled by the RSS-sensing repressor, CstR (22, 30, 31). Other genes that are induced by sulfide include (i) the terminal ubiquinol cytochrome *bd*-type oxidase, *cydAB* (*A1S_1433-1434*), as shown previously in E. coli and M. tuberculosis (9, 28), (ii) *A1S_0169-0171*, the middle gene encoding a candidate outer membrane localized copper transporter, OprC (32), and (iii) *A1S_3085-3086*, encoding a flavohemoglobin/nitric oxide dioxygenase (*hmp*) and a 4Fe-4S-containing nitric oxide-sensing transcriptional repressor (*nsrR*) (33), also upregulated in S. aureus treated with Na_2_S (29).

**FIG 3:**
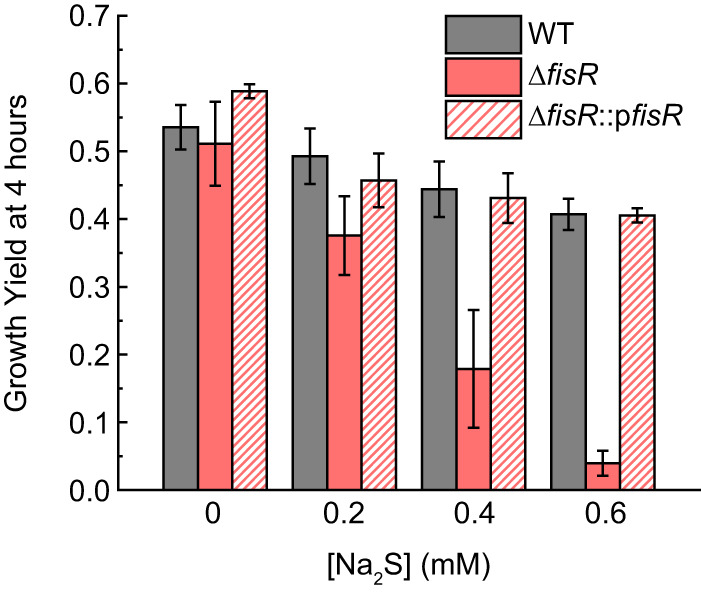
Growth yield after addition of Na_2_S. The growth yield at 4 h of WT, Δ*fisR*, and Δ*fisR*::p*fisR* strains with or without the indicated addition of Na_2_S at *t* = 0 is shown. See Fig. S2A to C in the supplemental material for full growth curves.

The *A1S_1708-1711* operon was of particular interest since the sulfide-dependent induction of these genes is not dependent on BigR (Fig. 2B) nor are they BigR-regulated genes, as determined by a transcriptome sequencing (RNA-seq) experiment that compares gene expression in the Δ*bigR* and WT strains (Fig. 1D; Table S1A). These data suggest an alternative mode of sulfide-inducible regulation of *A1S_1708-1711*. Upstream of this operon and transcribed in the opposite direction is *A1S_1707*, which encodes a canonical σ^54^-transcriptional activator, harboring an N-terminal regulatory domain, a central AAA+ ATPase domain, and a C-terminal DNA-binding domain (26, 34); further, the promoter upstream of *A1S_1708* is predicted to be a σ^54^-dependent promoter. We denote *A1S_1707* as *fisR* (Fis family transcriptional regulator), named after the sulfide-inducible σ^54^-dependent transcriptional activator previously characterized in *Cupriavidus* spp. (26). To test the hypothesis that FisR is a transcriptional activator of the *A1S_1708-1711* operon, we created a deletion strain of *fisR* and determined the ability of this strain to grow when challenged with exogenous sulfide (Fig. 3; Fig. S2A to C), while measuring expression of the BigR-regulated genes (*A1S_1876-1878*) and the *A1S_1708-1711* operon by qRT-PCR (Fig. 2C). The Δ*fisR* strain exhibits a pronounced growth lag relative to the Δ*bigR* and WT strains that becomes more severe with increasing exogenous sulfide (Fig. 3; Fig. S2). The sulfide-dependent induction of *A1S_1708-1711* is completely ablated in the Δ*fisR* strain (Fig. 2C) but not in the complemented strain (Fig. 2D); further, the sulfide-induced growth phenotype of the Δ*fisR* strain is also complemented in the Δ*fisR*::p*fisR* strain to WT growth levels (Fig. 3; Fig. S2A to C).

10.1128/mBio.01254-20.2 FIG S2 Growth of A. baumannii with addition of Na_2_S (related to Fig. 3). (A to C) Growth of WT, Δ*fisR*, Δ*fisR*::p*fisR*, Δ*fisR* Δ*bigR*, and Δ*fisR* Δ*bigR* mTn7(*bigR*) strains with the addition of 0.2 mM (A), 0.4 mM (B), 0.6 mM (C) Na_2_S. For clarity, only WT untreated is shown. (D) Growth of WT, Δ*bigR*, and Δ*bigR* mTn7(*bigR*) strains with or without the addition of 0.2 mM Na_2_S. Statistical significance was established using a paired *t* test relative to WT+Na_2_S [*, *P ≤ *0.05 for Δ*fisR*; **, *P ≤ *0.01 for Δ*fisR* Δ*bigR*; ***, *P ≤ *0.001 for Δ*fisR* Δ*bigR* mTn7(*bigR*)]. Download FIG S2, PDF file, 0.3 MB. Copyright © 2020 Walsh et al. 2020 Walsh et al. This content is distributed under the terms of the Creative Commons Attribution 4.0 International license.

Genes with reduced expression in the WT strain in the presence of exogenous sulfide are, as expected, largely limited to those genes associated with the uptake of other forms of sulfur (Fig. 1C; Table S1A). These include ABC transporters predicted to function in the uptake of the major organic sulfonate taurine (*tau*; *A1S_1442-1445*) and inorganic sulfate/TS (*A1S_2531-2536*). In addition, we observed repression of a putative transporter for polar amino acids glutamate and aspartate (*A1S_1396-1399*), whose substrate specificity has not yet been determined. Interestingly, the taurine and sulfate/thiosulfate transporters and a putative generalized alkanesulfonate transporter (*ssu*; A1S_0026-0030) are strongly upregulated by exogenous H_2_O_2_, some or all of which are under transcriptional control of the H_2_O_2_ sensor OxyR (35). These findings suggest that cells repress the uptake of other forms of sulfur when inorganic sulfide becomes available, while upregulating the uptake of these other forms of sulfur to protect against the effect of oxidative damage.

### BigR controls the expression of a secondary H_2_S/RSS-sensing and detoxification system.

The genomic BigR regulon was mapped by comparing transcriptomic profiles of the Δ*bigR* strain to that of a chromosomally complemented strain encoding a mutant allele of BigR that lacks the persulfide-sensing dithiol pair [C34, C100; Δ*bigR* mTn7(*bigR^C34A/C100A^*)] (Fig. 1E; Table S1A) (24, 36). These data suggest that in addition to *yeeE/yedE/pdo2*-encoding sulfide detoxification operon found previously in *Serratia* spp. (37), genes predicted to be associated with assembly of the type 1 chaperone-usher pilus involved in epithelial cell adhesion and biofilm formation (*A1S_1508-1510*) (38), an uncharacterized short-chain dehydrogenase (*sdr; A1S_0087*), and *A1S_1548-1549* may also be direct targets of BigR regulation, a finding consistent with the presence of candidate BigR operators upstream of these genes. Inspection of the qRT-PCR data obtained for the Δ*fisR* strain reveals that endogenous expression of the primary BigR-regulated operon (*A1S_1878-1876*) is ∼10-fold higher than the untreated WT strain (Fig. 2C). This suggests that under these growth conditions, there is significant endogenous H_2_S/RSS that FisR is sensing (Fig. 1B), and when FisR is absent, i.e., in the Δ*fisR* strain, this leads to endogenous induction of BigR-regulated genes to a level comparable to that of the Δ*bigR* strain (Fig. 2B).

As expected for a secondary or backup (per)sulfide sensing and detoxification system, the Δ*bigR* strain and complemented Δ*bigR* mTn7(*bigR*) strains grow indistinguishably from the WT strain in the absence or presence of exogenous sulfide (Fig. S2D); however, the double Δ*fisR* Δ*bigR* mutant grows better than the Δ*fisR* strain, while the complemented Δ*fisR* Δ*bigR* mTn7(*bigR*) strain grows even better than the uncomplemented strain (Fig. S2A to C). These experiments suggest that unregulated expression of the BigR-regulated genes in a Δ*bigR* strain protects A. baumannii against sulfide toxicity when FisR is not present and that BigR itself may also function as a persulfide “sink” under these conditions. These findings are consistent with a proteomics analysis of Δ*fisR* lysates, which reveals detectable BigR-regulated PDO2, in striking contrast to the WT strain (see below).

Thiol and persulfide profiling of mid-log-phase Δ*bigR* and Δ*fisR* strains reveals that loss of the H_2_S/RSS-sensing activator (Δ*fisR*) or loss of the RSS-sensing repressor (Δ*bigR*) both perturb organic LMW thiol persulfide abundance relative to the WT strain in untreated cells, with virtually no impact on total thiol levels (Fig. S3A and B). In both cases, GSSH levels are most significantly decreased, as expected from the expression of PDO2 in each strain as observed transcriptomically (Fig. 2), which is anticipated to specifically oxidize GSSH to sulfite (39, 40). The addition of 0.2 mM Na_2_S to these strains simply increases cellular persulfide levels, with the Δ*fisR* strain characterized by an organic persulfide content that is indistinguishable from those concentrations found endogenously in the WT (Fig. S3C). We conclude that although FisR and BigR-regulated gene products clearly impact cellular abundance of organic persulfides, even under sulfide-stressed conditions, none reach a level that exceeds ∼2% of the total thiol pool, as found for CoASH persulfide.

10.1128/mBio.01254-20.3 FIG S3 Metabolomic profiling of RSS in WT, Δ*bigR*, and Δ*fisR* strains. (A and B) Endogenous levels of LMW thiols (A) and organic persulfides, TS, and H_2_S (B). (C) Levels of organic persulfides, TS, and H_2_S 60 min after the addition of 0.2 mM Na_2_S. Dashed lines represent endogenous levels for WT A. baumannii. Statistical significance was established using a paired *t* test (**, *P ≤ *0.01; *, *P ≤ *0.05, ns; not significant). The significance indicated above each point is relative to the WT under the same condition with significance between Δ*bigR* and Δ*fisR* strains indicated below data points with brackets. WT RSS data are reproduced here from Fig. S1 to facilitate comparison with the mutant strains shown. Download FIG S3, PDF file, 0.2 MB. Copyright © 2020 Walsh et al. 2020 Walsh et al. This content is distributed under the terms of the Creative Commons Attribution 4.0 International license.

**FIG 4:**
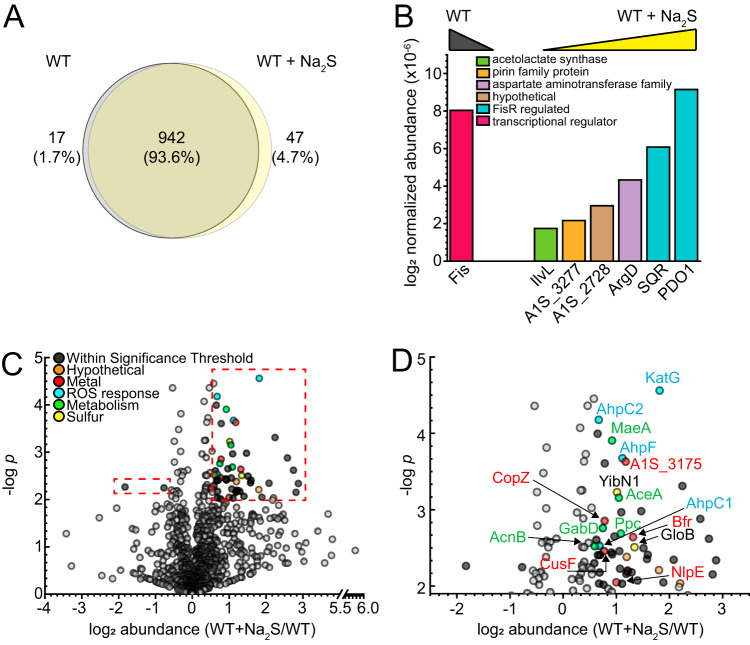
LC-MS/MS proteomics analysis of WT A. baumannii. Protein profiles of untreated (WT) versus 0.2 mM Na_2_S-treated (WT+Na_2_S) A. baumannii cells from four biological replicates are shown. (A) Venn diagram of proteins detected at least twice in four biological replicates. (B) Proteins that are identified only in untreated WT or WT+Na_2_S conditions in all four replicates and color-coded according to annotated function. (C) Volcano plot for proteins detected at least two times in four biological replicates. The significance threshold was set at *P* < 0.01, and the fold change in protein abundance was set at >1.5 as enclosed in the red dashed-line boxes. Circles corresponding to proteins within this significance threshold are shaded according to the annotated function. (D) Expanded view of proteins within the significance threshold in the red dashed-line boxes in panel C.

10.1128/mBio.01254-20.4 FIG S4 Proteomic analysis for WT and Δ*fisR* strains (relates to Fig. 4 and Fig. S6). (A to D) Histogram plots of the distribution of normalized abundance for all proteins detected in WT (A), WT + 0.2 mM Na_2_S (B), Δ*fisR* (C), and Δ*fisR* + 0.2 mM Na_2_S (D) strains. The topmost 50% abundant proteins are indicated with a red dashed line with the bars shaded red. Download FIG S4, PDF file, 0.3 MB. Copyright © 2020 Walsh et al. 2020 Walsh et al. This content is distributed under the terms of the Creative Commons Attribution 4.0 International license.

### Global proteomics profiling of wild-type *A. baumannii* before and after addition of exogenous sulfide.

We next employed a standard label-free “bottom-up” proteomics analysis (41) to estimate the degree to which cellular abundance of specific proteins change under conditions of exogenous sulfide stress (Table S1B). Four biological replicates of soluble lysates (cytoplasm and periplasm) were obtained from mid-log-phase untreated (WT) and sulfide-treated (WT+Na_2_S) cells (Fig. 4; Fig. S4A and B). In total, 1,006 proteins were detected at least twice in the four replicates, with 47 proteins observed only in the WT+Na_2_S cells and 17 observed only in WT untreated cells (Fig. 4A). Consistent with the transcriptomic induction of the FisR-regulated genes, we observe high cellular abundance of the PDO1 and SQR, each of which rises to the top 50% of proteins detected (Fig. 4B; Fig. S4B) and which are not detected in untreated cells. On the other hand, the nucleoid associated, nonspecific DNA binding protein Fis (A1S_2186) is only observed in untreated cells (Fig. 4B). In addition to these, there are several proteins whose cellular abundance significantly changes upon treatment with exogenous sulfide as depicted in a standard volcano plot format (Fig. 4C and D). Of interest are the increased abundance of major ROS detoxification enzymes, including a heme-cofactored catalase (KatG) and putative alkylhydroperoxidase subunits AhpF, AhpC1, and AhpC2, two universal stress proteins (Usps) (42), as well as an uncharacterized multidomain sulfurtransferase (A1S_2708). Interestingly, AhpC2 is among the most abundant nonribosomal proteins found in A. baumannii lysates among two AhpC and five AhpF proteins encoded by the A. baumannii genome (35). Furthermore, AhpC1 contains a single Cys active site, in contrast to AhpC2, which when sulfenylated in M. tuberculosis is known to react with H_2_S to form a persulfidated Cys; this persulfidated intermediate is highly active in persulfide transfer to a model RSH acceptor (18). We note that although some ROS enzymes have been shown to process (catalase) H_2_S or biosynthesize (superoxide dismutase) RSS, there is no evidence to date to suggest these reactions take place *in vivo* (43). In fact, we find the cellular responses to Na_2_S and H_2_O_2_ are largely independent of one another (Fig. S5) (35). This suggests either that OxyR does not respond directly to sulfane sulfur in A. baumannii as previously observed in E. coli (44) or that the induction of the OxyR regulon under these conditions is weak compared to the robust H_2_O_2_ response. As a result, only a few members of the regulon are induced at or above our detection limits.

10.1128/mBio.01254-20.5 FIG S5 Comparison of the proteomic and transcriptomic response between Na_2_S and H_2_O_2_ treatment of A. baumannii. (A to C) Proteomic and transcriptomic response to Na_2_S compared to the transcriptomic response for WT + H_2_O_2_ (A), Δ*oxyR* (B), and Δ*oxyR* + H_2_O_2_ (C) treatments. Transcriptomic data for H_2_O_2_ treatment, Δ*oxyR*, and Δ*oxyR* + H_2_O_2_ are taken from a previously published study (35). Download FIG S5, PDF file, 0.3 MB. Copyright © 2020 Walsh et al. 2020 Walsh et al. This content is distributed under the terms of the Creative Commons Attribution 4.0 International license.

10.1128/mBio.01254-20.6 FIG S6 Proteomic analysis for Δ*fisR* A. baumannii. Protein profiles of an untreated Δ*fisR* strain versus a 0.2 mM Na_2_S-treated Δ*fisR* strain (Δ*fisR*+Na_2_S) A. baumannii cells from four biological replicates are shown. (A) Venn diagram for proteins detected at least two times in four replicates. (B) Proteins that are only identified in Δ*fisR* or Δ*fisR*+Na_2_S conditions in all four replicates. (C) Volcano plot for proteins detected at least two times among four biological replicates. The significance threshold was set at *P* < 0.01, and fold change in protein abundance was set at >1.5, as shown in the red dashed-line boxes. Circles within this significance threshold are colored according to legend based on annotated function. (D) Expanded view of proteins within the significance threshold found in red boxes in panel C with same coloring scheme and gene products identified based on database annotations or locus tags. Download FIG S6, PDF file, 1.4 MB. Copyright © 2020 Walsh et al. 2020 Walsh et al. This content is distributed under the terms of the Creative Commons Attribution 4.0 International license.

**FIG 5:**
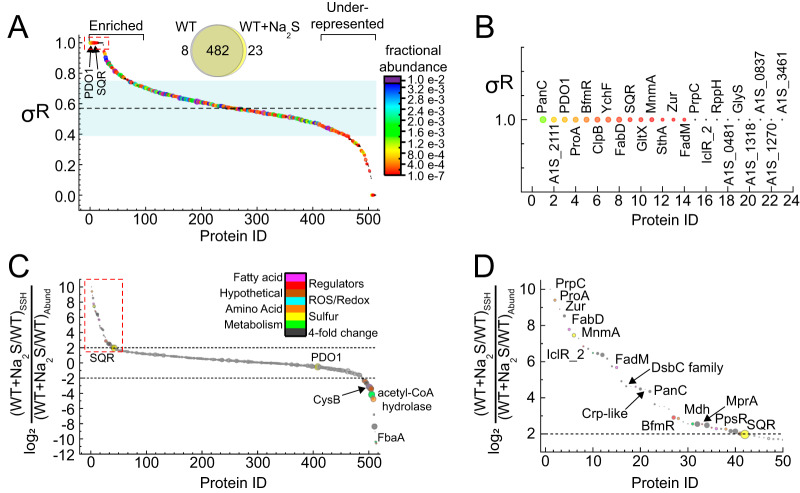
Proteome protein *S*-sulfuration profiling in WT A. baumannii. (A) Plot of σ^R^ versus protein ID, arbitrarily arranged from left to right according to σ^R^ (primary sort) and fractional abundance in WT+Na_2_S (secondary sort). Each symbol represents a single protein and is colored and sized according to the fractional abundance of that protein in WT+Na_2_S cells determined without enrichment. The dashed, horizontal line represents the mean σ^R^ (0.58), and the blue shaded area represents one standard deviation of the mean. The red box indicates the section expanded in panel B. σ^R^ is defined as the sum of all cysteine peptides in WT+Na_2_S over the total cysteine peptides in both WT and WT+Na_2_S, i.e., Σ(WT+Na_2_S)/[Σ(WT+Na_2_S)+Σ(WT)]. (B) Expanded view of red-boxed data in panel A highlighting proteins with a σ^R^ of 1, with proteins labeled as annotated gene products or locus tags. (C) Plot of protein *S*-sulfuration normalized to protein abundance. Each symbol represents a single protein and is sized according to the change in fractional abundance (WT+Na_2_S/WT), i.e., a larger circle represents a large increase in fractional abundance in WT+Na_2_S versus untreated WT cells. The horizontal dashed lines correspond to a 4-fold normalized increase or decrease in protein *S*-sulfuration. Persulfidated peptides with a 4-fold change in normalized abundance are shaded according to function based on gene annotations. The *red* box represents the section of plot expanded in panel D. (D) Expanded view of the red-boxed data in panel C highlighting proteins with a >4-fold increase in persulfidation, with proteins labeled as annotated gene products or locus tags.

10.1128/mBio.01254-20.7 FIG S7 (A) Sequence motif analysis of persulfidation sites using pLogo applied to all persulfidated cysteines identified in our data set relative to all cysteine containing proteins in the genome. The numbers of aligned foreground and background sequences are 798 and 10,390, respectively. The red horizontal bars correspond to *P = *0.05. (B) Same motif analysis applied only to high σ^R^ (above one standard deviation; not normalized to change in cellular abundance) sites. The number of foreground sequences is 106. (C) Proteome protein persulfidation normalized to protein abundance (related to Fig. 5). Each symbol represents a single protein arbitrarily arranged from left to right with proteins color-coded according to σ^R^, with red being greater than (σ^R^ ≥ 0.76) or blue being less than (σ^R^ ≤ 0.40) one standard deviation of the mean σ^R^ value (0.58). The dashed, horizontal lines represent a 4-fold increase or decrease in protein persulfidation. Selected proteins of interest are annotated. Download FIG S7, PDF file, 1.4 MB. Copyright © 2020 Walsh et al. 2020 Walsh et al. This content is distributed under the terms of the Creative Commons Attribution 4.0 International license.

In addition, the cellular abundance of several proteins involved in metal homeostasis are also changed (Fig. 4C and D). We observed increased abundance of CusF and CopZ, which are the periplasmic and cytoplasmic copper chaperones, respectively, and NlpE, an outer membrane lipoprotein that senses outer membrane stress, and is also involved in the response to periplasmic Cu stress (45). OprC, which is projected to bring Cu into the periplasm, is also transcriptionally upregulated by sulfide stress (Fig. 1C). In addition, two ferritin-like proteins, a heme-cofactored bacterioferritin (BfrB, A1S_3175) and a bacterial ferritin (FtnA, A1S_0800), both of which mineralize Fe for storage, are increased in the proteome (Fig. 4D and C) and are transcriptionally upregulated ∼3-fold by exogenous sulfide (Table S1A). These Fe storage proteins show high sequence similarity to BfrB and FtnA (formerly BfrA) in P. aeruginosa (46). In Pseudomonas aeruginosa FtnA exhibits constitutive expression during the exponential phase and Fe-dependent expression during the stationary phase, whereas BfrB is increased under excess Fe regardless of the growth phase (47, 48). Upregulation of FtnA and BfrB in A. baumannii under Na_2_S may be indicative of conditions of excess bioavailable Fe but further studies are required to substantiate this. Taken together, these data suggest that exogenous sulfide impacts transition metal homeostasis in cells.

We also carried out a proteomics analysis of the Δ*fisR* strain before and after addition of sulfide (Fig. S4C and D, Fig. S6, and Table S1C) in an effort to understand the impact of the weakly constitutive expression of the BigR-regulated operon in this strain (Fig. 2C), which gives rise to lower endogenous levels of GSSH (Fig. S3B). These findings make the prediction that the BigR-regulated PDO2 would be active in cells and detectable in the proteome under both conditions, but only in the Δ*fisR* strain. This is exactly what is observed, with no significant change in cellular abundance following the addition of Na_2_S to the Δ*fisR* strain (Fig. S6C). Aside from this, many of the perturbations in the global proteome in the WT versus Δ*fisR* strains are common to both strains, as expected (Fig. S6). A few proteins of note whose change in abundance with Na_2_S is significantly more in Δ*fisR* than WT include a ferric siderophore receptor protein (BfrD1, A1S_0474), a glutathione-dependent disulfide bond oxidoreductase (A1S_1411), and a nitrite/sulfite reductase (Sir, A1S_2846) (Fig. S6D and Table S1C).

### Mapping of proteome persulfidation sites.

We previously developed a global enrichment-reduction strategy to identify cysteines in the proteome that are subject to persulfidation in the absence or presence of the exogenous Na_2_S (11). Briefly, in this method, like others (49, 50), proteins in a lysate that harbor either a thiol or persulfide are enriched by alkylation with a biotinylated iodoacetamide (bioIAM) and adsorbed to neutravidin beads. Persulfide-containing peptides are eluted by reduction with TCEP [Tris(2-carboxyethyl)phosphine hydrochloride], capped with IAM, and subjected to liquid chromatography-tandem mass spectrometry (LC-MS/MS). This peptide fraction is therefore enriched for peptides from proteins that are persulfidated in cells before or after addition of exogenous sulfide, reflected in the parameter σ^R^, which is a measure of the extent to which a particular Cys residue is more heavily persulfidated in cells 30 min after the addition of sulfide versus prior to addition of sulfide (Fig. 5A). A σ^R^ value of 1.0 identifies peptides that are not persulfidated prior to the addition of Na_2_S (23 proteins), a σ^R^ = 0.5 indicates equal peptide counts prior to and after the addition of Na_2_S, and a σ^R^ value approaching 0 indicates peptides that are only persulfidated in the absence of exogenous sulfide (8 proteins).

We identify 513 proteins or 13% of the proteome that harbor at least one persulfidated thiol, with the vast majority of these (95%) persulfidated in both unstressed and stressed cells (Fig. 5; Table S1D). This finding is as expected from previous persulfidation profiling studies of S. aureus (11) and E. faecalis (B. Walsh, unpublished results) and suggests that the proteome provides a readily tapped reservoir of bioactive sulfane sulfur (11). These proteins map to several cellular processes, including fatty acid and amino acid biosynthesis and pyruvate, pyrimidine, and sugar metabolism. In an effort to identify a persulfidation motif, all modified peptides were analyzed by the pLogo motif analyzer program (Fig. S7A). Although this analysis did not reveal conserved residues on either side of the persulfidated cysteine, hydrophilic residues are overrepresented, while hydrophobic and additional cysteine residues are underrepresented. This result is consistent with other studies, for protein persulfidation or *S-*sulfenylation but perhaps not for *S-*nitrosylation or electrophile-sensing cysteine thiolates (51, 52). When we examines only peptides with high σ^R^, we still did not observe a consensus persulfidation motif, but the pattern of hydrophilicity vanished (Fig. S7B).

We next recast our σ^R^ plot in a way that is normalized to a change in protein cellular abundance as a ratio (log_2_) of ratios of the persulfidated peptide yield versus change in cellular abundance in the presence versus absence of exogenous sulfide (Fig. 5C; see also Fig. S7C and Table S1D in the supplemental material). Although this approach does not measure the actual fraction of persulfidated cysteine versus thiol in any one peptide, the normalized fold change in abundance of a persulfidated peptide must be indicative of an increase in this fraction. We found that the vast majority of the proteins with σ^R^ values greater than (σ^R^ ≥ 0.76) or less than (σ*^R^* ≤ 0.40) one standard deviation from the mean σ^R^ value (0.58) are found in the wings of the normalized abundance plot as well (Fig. S7C) and correspond to a normalized change in persulfidation versus thiol status of greater than or less than 4-fold. Prominent exceptions to this trend are the FisR-activated PDO1 and SQR, which are only detected in the sulfide-treated cells and are thus characterized by a large increase in cellular abundance, and correspond to the group of 23 proteins with a σ^R^ of 1 (Fig. 5B). In fact, four of the five Cys in PDO1 were detected as persulfidated, while two Cys in SQR were modified, including the presumed active site thiol, C248, expected to carry a persulfide as a catalytic intermediate (31). Cys-containing peptides found to be significantly enriched in persulfidation status are of interest since they represent protein targets for which persulfidation may be regulatory (Fig. 5D). Included in this group of enzymes is MnmA, the tRNA 2-thiouridylase, which incorporates s^2^U substitution at U34 in tRNAs. The persulfidated Cys is the resolving Cys in a two-Cys mechanism that donates the sulfur atom for thio-insertion (53, 54).

In addition, there are five transcriptional regulators found in this group of proteins, and these include the zinc uptake repressor, Zur (55); the A. baumannii acetate operon repressor, IclR2; a cell-abundant catabolite regulatory protein (Crp)-like protein (A1S_1182); and the biofilm response regulator, BfmR (Fig. 5B). BfmR is part of the BfmRS two-component system and master regulator of biofilm formation and desiccation tolerance that controls the expression of the Csu chaperone-usher assembly system which produces the cell surface pili required for adherence to abiotic surfaces (56). The persulfidated Cys in BfmR is found in the receiver domain and close to the site of phosphorylation (D58), which regulates DNA binding (57). The persulfidated Cys in Zur is conserved in E. coli Zur as C17 and is located in the α1 helix, close to the bound DNA within the N-terminal DNA binding domain (58). The impact of persulfidation on IclR2 and Crp function is also not known, nor has A. baumannii Crp been functionally characterized in any detail (59). However, A. baumannii Crp is quite closely related to global regulators Crp from E. coli (50% identical) and Vfr from P. aeruginosa (54% identical), with the conserved Cys opposite the ligand binding pocket, which for E. coli Crp is cyclic AMP (cAMP) (60). Glutathione is an allosteric activator of Vfr transcriptional upregulation of the type III secretion system in P. aeruginosa (61); the same is true of the global regulator PrfA in Listeria monocytogenes (62, 63). The small molecule activator of A. baumannii Crp-like protein is unknown.

## DISCUSSION

This study presents a comprehensive analysis of the adaptive response of the major human pathogen A. baumannii to exogenous hydrogen sulfide stress as a model for intracellular sulfide accumulation and thus identifies essential features of sulfide/RSS homeostasis. Although it is not known under what particular niche A. baumannii might encounter exogenous sulfide, emerging evidence suggests that endogenous sulfide/RSS accumulation may protect a wide range of bacterial pathogens against clearance by the immune system, while reducing its susceptibility to antibiotics (6, 8–10, 64, 65); in addition, increased cellular H_2_S may characterize cells in biofilms at low O_2_ availability (66). We show here that A. baumannii contains the LMW thiols GSH, cysteine, HCys, and CoASH and detectable levels of their corresponding thiol persulfides. The addition of exogenous sulfide results in assimilation of this inorganic sulfide as organic thiol persulfides namely, GSSH, cysteine persulfide, as well as the inorganic RSS, TS. While 3MST is reported as a source of intracellular H_2_S in E. coli (5, 8), we found that the deletion of 3MST in A. baumannii results in significantly decreased thiol persulfides with little detectable impact on measured H_2_S concentrations. This suggests that any H_2_S production by 3MST is readily converted to thiol persulfides, perhaps by FisR-regulated SQR, which may function as cellular antioxidants to reduce immune system clearance during infection (6). In addition, intracellular production of H_2_S or RSS suggests a need for tight regulation of these species to avoid H_2_S toxicity while not overclearing RSS, which impacts virulence in a variety of infection models in S. aureus and in E. faecalis (6, 11, 23).

**FIG 6:**
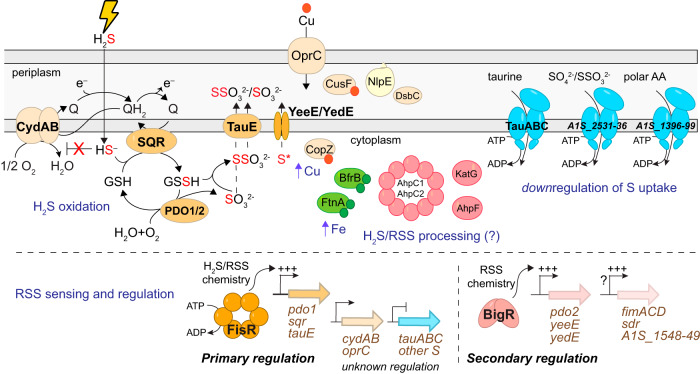
Cartoon summary of the findings presented here. In response to increased cellular sulfide, a program of H_2_S oxidation ensues (left), where sulfide is oxidized to sulfite by the combined action of the two FisR-regulated gene products, SQR and PDO1, with the intermediacy of an LMW thiol persulfide RSSH (GSSH is shown); thiosulfate (SSO_3_^2–^) may be formed by the uncatalyzed reaction of sulfite with RSSH as shown (39). Sulfite and/or thiosulfate may be exported through the exporter TauE as indicated (78), while an unknown form of sulfur (S*) is transported by YeeE/YedE (37). Meanwhile, the ubiquinol oxidase CydAB is upregulated and is refractory to inhibition by sulfide (9, 28); the activities of SQR and CydAB are predicted to be linked though the ubiquinone pool (Q) (14). Evidence of increased bioavailable Cu (red spheres) derives from increased Cu importer expression (OprC) (41), increased expression of major Cu chaperones, and NlpE, linked to Cu(II)-linked lipoprotein stress and/or persulfidation of periplasmic thiolates (DsbC) (45). Increased bioavailable Fe (green spheres) is suggested by increased cell abundance of bacterioferritin (BfrB) and bacterial ferritin (FtnA). We also observed increased cellular concentrations of ROS enzymes (pink symbols). Finally, we observed downregulation of at least three ABC transporters involved in uptake of sulfur-containing molecules (other S; locus tags indicated), all via unknown regulatory mechanisms. Both primary and secondary H_2_S/RSS sensing and detoxification systems regulated by FisR and BigR, respectively, are shown below the gray dashed line. FisR is transcriptionally activated (+++) as a result of increased cellular H_2_S/RSS, while BigR-mediated RSS chemistry (36) drives transcriptional derepression (+++) of BigR-regulated genes. Genes indicated by a question mark (?) are candidate direct BigR targets.

We find that A. baumannii encodes primary and secondary regulatory mechanisms responsible for sulfide detoxification and RSS clearance (Fig. 1 and 6). The primary mechanism is transcriptionally regulated by FisR, a σ^54^-dependent transcriptional activator, and detoxifies sulfide via SQR, PDO1, and a putative thiosulfate/sulfite effluxer, TauE, with the role of the homoserine dehydrogenase not known. The secondary mechanism involves transcriptional regulation by the ArsR-family RSS-sensing repressor BigR (36), whose operon includes a second PDO2 and two putative transmembrane proteins proposed previously in *Serratia* spp. to function in the transport of sulfur-containing molecules, and associated with the biosynthesis of the antibiotic prodigiosin (37). This secondary system is only active upon deletion of the repressor BigR, or upon deletion of the primary activator FisR (Fig. 2), suggesting this regulon acts as a backup system but only when FisR is unable to activate its regulon. Considering that A. baumannii FisR does not conserve the regulatory domain cysteines previously shown to contribute to sulfide-dependent gene expression in *Cupriavidus*, it is unclear how FisR senses H_2_S/RSS in A. baumannii (26). It is possible that FisR and BigR recognize a different small molecule or bind the same inducer but with different sensitivities. BigR, however, clearly performs thiol oxidative chemistry with organic persulfides (36).

In addition to the sulfide detoxification mechanisms, we have analyzed both the transcriptional and proteomic response of A. baumannii to exogenous sulfide (Fig. 6). This includes transcriptional upregulation of the alternative cytochrome oxidase *cydAB*, which is refractory to inhibition by H_2_S (10, 28), coupled with downregulation of the ABC transporters predicted to function in the uptake of taurine and TS/sulfate. This allows the organism to maintain respiratory flux and redox balance in the presence of H_2_S while limiting the uptake of other sulfur sources to utilize the inorganic sulfide at hand (9). Our proteomic analysis reveals increased abundance of ROS detoxification enzymes that are largely independent from the response of A. baumannii to H_2_O_2_, indicating that these enzymes may have roles in sulfide homeostasis in addition to the ROS response. Exogenous sulfide also appears to induce a high-Fe and high-Cu response in A. baumannii that is incompletely understood (Fig. 6). In addition, we noted significant increases in cellular abundances of metabolic enzymes aconitase, perhaps facilitated by apparently increased bioavailable Fe and S, isocitrate lyase, succinate semialdehyde dehydrogenase, and malate synthase, all of which suggests enhanced metabolic flux through the glyoxylate shunt (GS; Fig. S8) (67, 68). The GS was previously shown to be critical for the growth of M. tuberculosis within the infected host (69) and for maintenance of Fe homeostasis in P. aeruginosa (68). Indeed, persulfidation of the acetate operon repressor IclR2 may function as a regulatory mechanism that controls flux through the GS under these conditions (70); however, GS and its regulation has not been extensively studied in A. baumannii.

10.1128/mBio.01254-20.8 FIG S8 Metabolic hits in proteomic analysis of A. baumannii with Na_2_S. A. baumannii encodes a tricarboxylic acid cycle and glyoxylate shunt pathway. Proteins significantly changed (>1.5-fold, *P* < 0.01) in the proteome upon addition of Na_2_S are indicated by boldface text and blue boxes. The green box indicates genes significantly changed just outside our significance threshold (>1.3-fold change and *P* < 0.05). Italicized locus tags indicate proteins that were identified in the proteomic analysis but are not significantly changed in cellular abundance. Circles represent proteins identified as persulfidated in the proteome, where red circles indicate a >4-fold change in persulfidation status (normalized to protein abundance) and blue indicates a <4-fold change. The red arrows indicate the glyoxylate shunt. Download FIG S8, PDF file, 0.3 MB. Copyright © 2020 Walsh et al. 2020 Walsh et al. This content is distributed under the terms of the Creative Commons Attribution 4.0 International license.

We found that ∼13% of the proteome in A. baumannii harbors persulfidated cysteines and the extent of persulfidation changes significantly with exogenous sulfide with little or no change in protein abundance for a number of these targets (Fig. 5). This, together with our sequence motif analysis, suggests that many of these modified cysteines must be surface exposed and potentially function as a sink for cellular sulfane sulfur, a finding consistent with previous studies in S. aureus (11). However, the persulfidation status of five transcriptional regulators changes significantly (≥10-fold) under conditions of exogenous sulfide, and these therefore represent excellent candidate regulatory targets of protein *S-*sulfuration. Of particular interest is the master regulator of biofilm formation, BfmR, and a Crp (cAMP regulatory protein)-like regulator, each of which possess persulfidated Cys that may well be regulatory, given their physical positions within the known or predicted structures of these regulators (57, 71). In addition, the Crp activity in other organisms, e.g., L. monocytogenes, is directly regulated by glutathione binding (62, 63). Like BfmR, the Crp-like regulator in A. baumannii may also play a role in biofilm formation, specifically in the formation of the pellicle, a specialized form of biofilm characterized in Acetobacter aceti (72). In A. baumannii the cAMP phosphodiesterase CpdA was found to be crucial for pellicle formation (59) and in P. aeruginosa the Crp regulator, Vfr, transcriptionally regulates *cpdA* (73). In addition, a *cpdA* mutant A. baumannii strain exhibits decreased expression of the *csu* system that is regulated by BfmR positing a potential connection between these two regulatory systems (59).

The Crp-like regulator and BfmR in A. baumannii remain largely uncharacterized as to their mechanisms of regulation (57). Our data suggest that elevated H_2_S/RSS-dependent persulfidation may impact their transcriptional programs. Such a connection of sulfide homeostasis to the regulation of biofilm dynamics may also extend beyond the *csu* system, since BigR, directly or indirectly, regulates the expression of genes predicted to be associated with assembly of the type 1 chaperone-usher pilus (Fig. 1D) involved in epithelial cell adhesion and biofilm formation in other strains (38). We note that elevated H_2_S is sometimes associated with bacterial cells under anoxic or low O_2_ tension that persist deep in a biofilm, e.g., in cystic fibrosis sputum samples (66), which suggests that H_2_S/RSS-driven persulfidation of one or more master regulators may constitute a regulatory switch to dynamically control biofilm formation. This regulatory hypothesis is analogous to our previous findings in S. aureus where widespread persulfidation of global virulence regulators was shown to alter the composition of the secretome, thus impacting immune cell killing potential (11). Current studies are directed toward understanding how H_2_S/RSS biogenesis and the dual RSS sensing and clearance systems described here impact host virulence in mouse models of infection (6).

## MATERIALS AND METHODS

### Bacterial strains and growth.

Strains were grown in Luria-Bertani (LB) medium or on LB plates including 1.5% (wt/vol) agar. Antibiotics were included at the following concentrations: carbenicillin, 50 mg/liter for E. coli and 75 mg/liter for A. baumannii; tetracycline, 10 mg/liter; kanamycin, 40 mg/liter; chloramphenicol, 15 mg/liter; and sulfamethoxazole, 100 mg/liter. Each overnight culture was initiated from a single colony, followed by incubation at 37°C with shaking for 8 to 16 h. Overnight cultures were diluted 1:50 in fresh LB medium, followed by incubation for 1 h before inoculation at 1:100. For growth curves, stressors were added after the 1:100 dilution at the indicated concentrations, and growth was monitored based on the optical density at 600 nm (OD_600_) using a GENESYS 20 Visible Spectrophotometer (Thermo Scientific).

### Construction of plasmids, allelic exchange mutants, and genetic constructs.

All strains, plasmids, and oligonucleotides used in this study are listed in Table S2A, Table S2B, and Table S2C and D, respectively. DNA was amplified with 2× Phusion master mix (Thermo Fisher). All restriction enzymes were from NEB. pET3a-BigR was generated by amplification of *bigR* gene from wild-type A. baumannii ATCC 17978 genomic DNA and cloned into BamHI and NdeI sites via Gibson assembly (36). This vector was then used to construct BigR double Cys mutant pET3a-BigR^C34A/C100A^ using a standard mutagenesis protocol.

10.1128/mBio.01254-20.10 TABLE S2 (A) Bacterial strains used in this study. (B) Plasmids used in this study. (C) Primers used for construction of strains and plasmids. (D) Primers used in qRT-PCR experiments. Download Table S2, XLSX file, 0.02 MB. Copyright © 2020 Walsh et al. 2020 Walsh et al. This content is distributed under the terms of the Creative Commons Attribution 4.0 International license.

To construct Δ*fisR*::Tc, Δ*bigR*::Kn, and Δ*mstA*::Kn strains, 1,000-bp regions flanking the genes to be deleted were amplified from A. baumannii ATCC 17978 genomic DNA. For the Δ*fisR*::Tc strain, the excisable tetracycline resistance gene *tetA* was amplified from pLDP55; for the Δ*bigR*::Kn and Δ*mstA*::Kn strains, the excisable kanamycin resistance gene *aph* was amplified from pKD4 (74). The PCR products were assembled into pFLP2 digested with BamHI and KpnI using NEB HiFi to generate pLDP79, pLDP39, and pLDP81, respectively. After electroporating A. baumannii ATCC 17978 and selecting for kanamycin resistance (Kn^r^) or tetracycline resistance (Tc^r^), colonies were patched to screen for carbenicillin resistance (Carb^r^) and sucrose sensitivity (Suc^s^). Suc^s^ and PCR-confirmed merodiploids were counterselected on LB agar plates containing 10% sucrose and screened for Kn^r^/Tc^r^ and Carb^s^. *fisR* complementation vectors were constructed in pLDP29, a derivative of pWH1266 (75), digested with BamHI and KpnI. To construct the *bigR* complementation strains, the *bigR* promoter and gene were amplified from wild-type A. baumannii ATCC 17978, or the gene was amplified from pET3a-*bigR^C34A/C100A^*. DNA fragments were ligated into pKNOCK-mTn7-Amp digested with BamHI and KpnI by HiFi assembly. The mini-Tn*7* was introduced into the A. baumannii Δ*bigR*::Kn strain as previously described (76), using carbenicillin as selection and chloramphenicol to counterselect against E. coli. All strains were confirmed by PCR and checked for maintenance of the large conjugative plasmid pAB3 by resistance to sulfamethoxazole.

### Quantitative RT-PCR.

Overnight cultures of A. baumannii were diluted 1:50 in LB medium and grown for 1 h at 37°C. Cultures were then diluted 1:100 into 10 ml of LB medium and grown to an OD_600_ of 0.2, followed by the addition of Na_2_S to a final concentration of 0.2 mM, and then incubated for an additional 30 min. Samples were then centrifugation at 4°C, washed with cold phosphate-buffered saline (PBS), and cell pellets were stored at – 80°C. Pellets were thawed on ice and resuspended in 1 ml of TRI Reagent (catalog no. TR-118; Molecular Research Center). Resuspended cells were placed in tubes containing 0.1-mm silica beads (Lysing matrix B tubes, catalog no. 6911-100; MP Biomedicals) and lysed in a bead beater (Bead Ruptor 24 Elite; Omni) at a rate of 6 m/s for 45 s twice, with a 5-min cooling on ice between runs. Then, 200 μl of chloroform was added, followed by vigorous mixing and centrifugation for 15 min at 13,200 rpm. The top aqueous layer was removed to a new tube, and 70% ethanol was added at a 1:1 volume ratio. RNA purification was completed using the RNeasy minikit (catalog no. 74104; Qiagen) following DNase I treatment (catalog no. 79254; Qiagen). Next, 5 μg of total RNA was subsequently digested with the DNA-free kit (catalog no. AM1906; Ambion) and diluted 5-fold. First-strand cDNA was synthesized using random hexamers (Quanta Biosciences) and a qScript Flex cDNA synthesis kit (catalog no. 95049-100; Quanta Biosciences). Reactions contained 10 μl of 2× Brilliant III Ultra-Fast SYBR green QPCR master mix (catalog no. 600882; Agilent), 2 μl each of 2 μM PCR primers (Table S2D), 0.3 μl of 2 μM ROX reference dye, and 6 μl of diluted cDNA. Relative transcript amounts were measured using an MX3000P thermocycler (Stratagene) running SYBR green with a dissociation curve program and normalized to the amount of 16S rRNA. The thermal profile consisted of 1 cycle at 95°C for 3 min, followed by 40 cycles at 95°C for 20 s to 59°C for 20 s. Subsequently, a dissociation curve starting at 55°C and going to 95°C in 0.5°C increments with a dwell time of 30 s was applied to assess the specificity of the reactions. Fold changes were calculated from threshold cycle (*C_T_*) values by comparing the ratio of treated to nontreated wild-type cells after normalization to 16S rRNA. Two technical replicates of three biological replicates were measured for each condition, and the means ± the standard deviations (SD) are reported. Transcript amounts were compared using a Student *t* test.

### RNA sequencing.

RNA samples were prepared in the same way as for the qRT-PCR with samples collected after 10 min after the addition of 0.2 mM Na_2_S at an OD_600_ of 0.2. Samples were submitted to the Center of Genomics and Bioinformatics at Indiana University to conduct RNA-seq analyses. The RNA integrity number was determined with TapeStation before the rRNA was removed using a Ribominus transcriptome isolation kit (Invitrogen, catalog no. K1550), and a library was generated using a TruSeq stranded mRNA library prep kit (Illumina). The results of all RNA-seq experiments have been deposited in the GEO database under GenBank accession number GSE148264.

### Quantitation of cellular thiols and persulfides.

Cellular profiling of RSS was performed as previously described (29). Briefly, overnight cultures were inoculated 1:50 in LB medium for 1 h prior to inoculation at 1:100 into fresh LB medium, followed by growth at 37°C with shaking until the OD_600_ reached ∼0.2; Na_2_S was then added to the cell cultures at the indicated concentrations, and the cells were collected at the indicated times (time zero is before the addition of Na_2_S), washed with PBS, and stored at – 80°C until processing. The cell pellets were thawed, resuspended in 100 μl of labeling buffer (25 mM Tris [pH 8.0], 1 mM monobromobimane, and 0.1% Triton-X in 50% acetonitrile), and freeze-thawed six times using liquid N_2_ and a 37°C water bath. Cellular debris was pelleted by centrifugation, and the supernatant was transferred to a new 1.5-ml tube containing 100 μl of 15 mM methane sulfonic acid to terminate the labeling reaction. The samples were then filtered in a 0.22-μm spin filter prior to ultraperformance liquid chromatography/mass spectrometry, as previously described. Analysis was performed using MassLynx software, and quantitation was done by an internal standard synthesized as previously described (29).

### Proteomic analysis of wild-type and Δ*fisR A. baumannii* strains.

Cell cultures were prepared as described above for the RT-PCR experiments. Quadruplicate 10-ml cultures of wild-type and Δ*fisR* strains were grown aerobically at 37°C to an OD_600_ of 0.2. Cultures were then collected by centrifugation for untreated cells or treated with 0.2 mM Na_2_S for 30 min, followed by centrifugation. All cells were washed with cold PBS and stored at –80°C. Pellets were thawed on ice and resuspended in 600 μl of lysis buffer (25 mM HEPES [pH 7.4], 150 mM NaCl, 5 mM TCEP, EDTA-free protease inhibitor cocktail [1:500 dilution]). Resuspended cells were transferred to lysing matrix B tubes and lysed in a bead beater at a rate of 6 m/s for 45 s three times, with a 5-min cooling on ice in between runs. Samples were centrifuged at 13,200 rpm for 15 min at 4°C. The supernatant was transferred to a new 1.5-ml tube, and the protein concentration was quantified by a Bradford assay using a standard protocol. Portions (20 μg) of protein were dried in SpeedVac concentrator and resuspended in 8 M urea in 100 mM ammonium bicarbonate. To these solutions, 5 mM TCEP and 50 mM iodoacetamide were added for 45 min at room temperature to reduce and alkylate the cysteine residues. Proteins were then precipitated by 20% (vol/vol) trichloroacetic acid and let sit at –20°C for 1 h. Samples were centrifuged at 15,000 rpm for 20 min at 4°C with the supernatant removed. The pellets were washed with cold acetone and centrifuged twice at 15,000 rpm for 20 min at 4°C. Samples were dried in a SpeedVac concentrator, resuspended in 100 μl of 1 M urea in 100 mM ammonium bicarbonate, and digested overnight at 37°C with the addition of a 1:100 (wt/wt) ratio of trypsin. Peptides were desalted by using a C_18_ Omix Tip (Agilent Technologies) according to a standard protocol and analyzed as described below.

### Enrichment and identification of *S-*sulfurated proteins in *A. baumannii*.

Wild-type A. baumannii was grown as described above for proteomic analysis. Pellets from 400-ml cultures were resuspended in 1 ml of lysis buffer (25 mM MES [morpholineethanesulfonic acid], 200 mM NaCl [pH 6.0], degassed), and five freeze-thaw cycles were performed with liquid nitrogen and a 37°C water bath. The resuspended pellet was transferred to lysing matrix B tubes and lysed three times in a bead beater at a rate of 6 m/s for 45 s, with a 5-min cooling on ice between runs. Samples were centrifuged at 13,000 rpm for 15 min at 4°C. The supernatant was transferred to a new tube and spun at 13,000 rpm for 15 min at 4°C one additional time with the supernatant saved as the cell lysate. The protein concentration of the cell lysate was measured using a standard Bradford assay, and 8 mg of protein was used for the following workup, as previously described (11).

Briefly, lysates were buffer exchanged to 8 M urea in 100 mM ammonium bicarbonate and incubated with 2 mg of biotinylated iodoacetamide (b-IAM; Thermo Scientific) in the dark for 1.5 h at 25°C. The sample was then buffer exchanged to 1 M urea in 100 mM ammonium bicarbonate, digested with 60 μg of trypsin at 37°C overnight, desalted over a C_18_ column (Sep Pak; Waters), dried, and suspended in 1 ml of 100 mM ammonium bicarbonate. Then, 1 ml of neutravidin polyacrylamide resin (Thermo Scientific) was equilibrated with 100 mM ammonium bicarbonate and incubated with digested sample at room temperature for 1 h. The resin was then washed with 75 ml of 2 M NaCl in 100 mM ammonium bicarbonate, followed by a 15 ml of 100 mM ammonium bicarbonate wash at 37°C. The *S*-sulfurated peptides bound to the resin were then selectively removed by reduction with 1 ml of 20 mM TCEP at 37°C for 1 h, and the supernatant was collected as elution 1. This step was repeated, and the supernatant was collected as elution 2. The two elution samples were pooled and alkylated with 100 mM iodoacetamide in dark for 45 min at room temperature. The sample was then desalted with an Omix C_18_ tip (Agilent Technologies) and subjected to LC-MS/MS analysis as described below. Three biological replicates were used as treated (WT+Na_2_S) and untreated (WT) samples. Sigma ratios (σ^R^) were calculated for each protein using the total area for the cysteine peptides. The fold changes of *S-*sulfurated peptides (WT+Na_2_S/WT) were normalized to the protein abundance from data acquired in the proteomic analysis described above. The *S-*sulfurated peptides only detected in one condition (WT or WT+Na_2_S) or proteins not identified in our no-enrichment proteomic analysis were assigned peak areas equal to the lowest detected peptide or protein to eliminate division by zero during normalization.

### LC-MS/MS analysis of proteome and *S-*sulfurated proteins.

Peptides were injected into a nanoACQUITY high-pressure liquid chromatography (HPLC) system coupled to an Orbitrap Fusion Lumos mass spectrometer (Thermo Scientific, Bremen, Germany). The peptides were separated using a nanoACQUITY HPLC HHS T3 analytical column (75 μm × 150 mm, 1.8 μm) using an acetonitrile-based gradient (solvent A, 0% acetonitrile and 0.1% formic acid; solvent B, 80% acetonitrile and 0.1% formic acid) at a flow rate of 400 nl/min. A 90-min gradient was applied as follows: 0 to 0.5 min, 0 to 14% B; 0.5 to 72 min, 14 to 50% B; 72 to 74 min, 50 to 100% B; 74 to 77 min, 100% B; 77 to 78 min, 100 to 0% B; 78 to 90 min, isocratic flow at 0% B. The electrospray ionization was carried out with a nanoESI source at a 260°C capillary temperature and 1.8-kV spray voltage. The mass spectrometer was operated in data-dependent acquisition mode with mass range 350 to 2,000 *m/z*. The precursor ions were selected for MS/MS analysis in Orbitrap with 3-s cycle time using higher-energy collisional dissociation (HCD) at a 35% collision energy. The intensity threshold was set at 5e3. The dynamic exclusion was set with a repeat count of 1 and an exclusion duration of 30 s. The resulting data were searched against an A. baumannii database (UniProt UP000094982, 3,780 entries) in Proteome Discoverer 2.1. Carbamidomethylation of cysteine residues was set as a fixed modification. Protein N-terminal acetylation, oxidation of methionine, protein N-terminal methionine loss, protein N-terminal methionine loss and acetylation, and pyroglutamine formation were set as variable modifications. A total of three variable modifications were allowed. A trypsin digestion specificity with two missed cleavages was allowed. The mass tolerances for precursor and fragment ions were set to 10 ppm and 0.6 Da, respectively.

### Statistical rationale and bioinformatics analysis.

Proteins detected in fewer than two replicates were excluded from our statistical analysis. The statistical analysis of these data was completed using an unpaired, two-tailed Student *t* test with Welch’s correction. Functional information for the selected proteins were gathered from the National Center for Biotechnology Information (NCBI; https://www.ncbi.nlm.nih.gov/), BioCyc (https://biocyc.org/web-services.shtml), and Kyoto Encyclopedia of Genes and Genomes (KEGG; https://www.genome.jp/kegg/pathway.html) databases. Metabolism pathway information for the selected proteins were obtained from KEGG and pathway analysis of *S-*sulfurated proteins was performed using KEGG pathway mapper (https://www.genome.jp/kegg/mapper.html). Motif analysis of *S-*sulfuration sites was performed using pLogo (http://plogo.uconn.edu) (77). For each modified cysteine, a 21-amino-acid sequence containing the *S*-sulfurated cysteine, with 10 amino acids flanking sequences on either side of the Cys, was selected. A background data set was constructed similarly using all cysteines identified in the reference genome for A. baumannii ATCC 17978 (GenBank accession no. NZ_CP018664.1).

### Data availability.

All RNA-seq experiment results have been deposited in the GEO database under GenBank accession number GSE148264.
