# Supplementary figures and images for: The Response of Acinetobacter baumannii to Hydrogen Sulfide Reveals Two Independent Persulfide-Sensing Systems and a Connection to Biofilm Regulation
Source: mBio. 2020 Jun 23;11(3):e01254-20. doi: 10.1128/mBio.01254-20 (PMC7315123; doi:10.1128/mBio.01254-20)

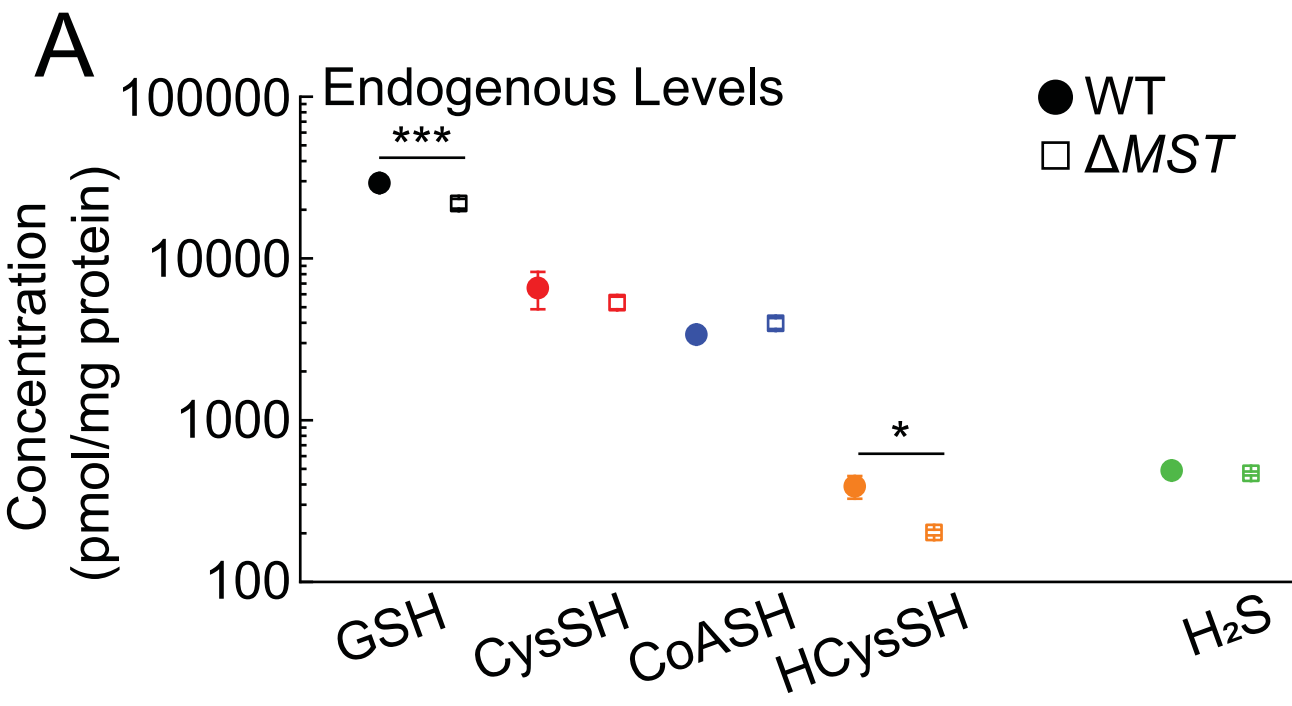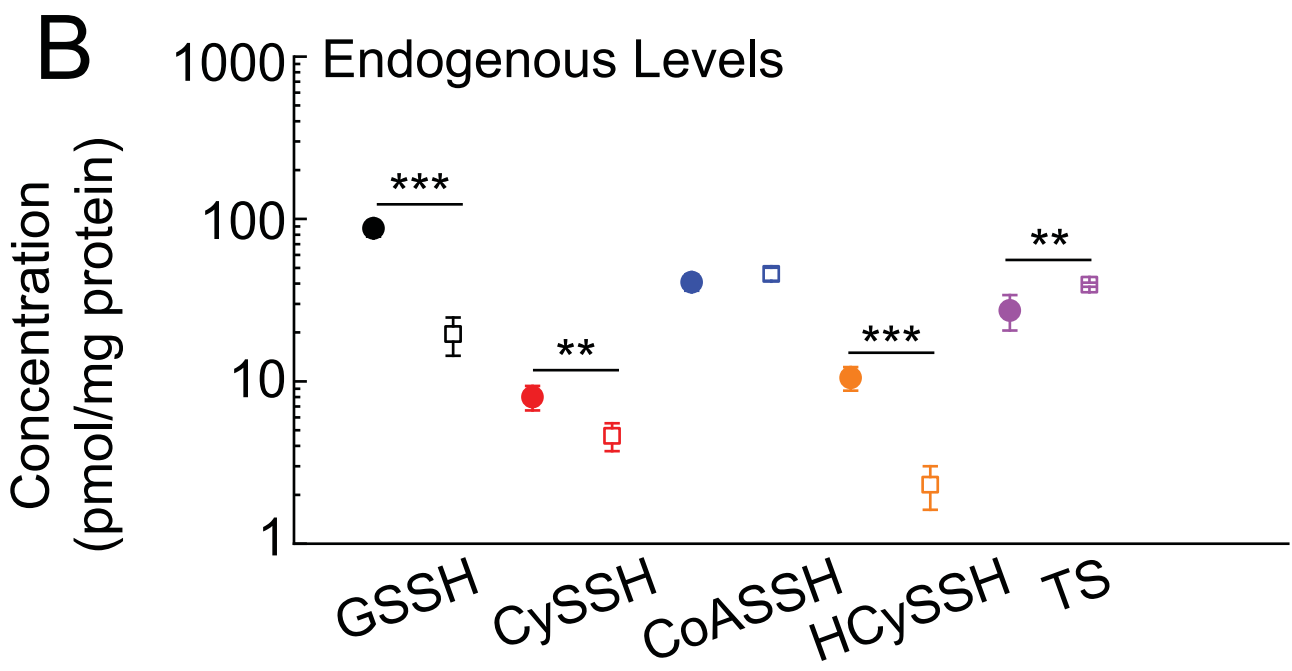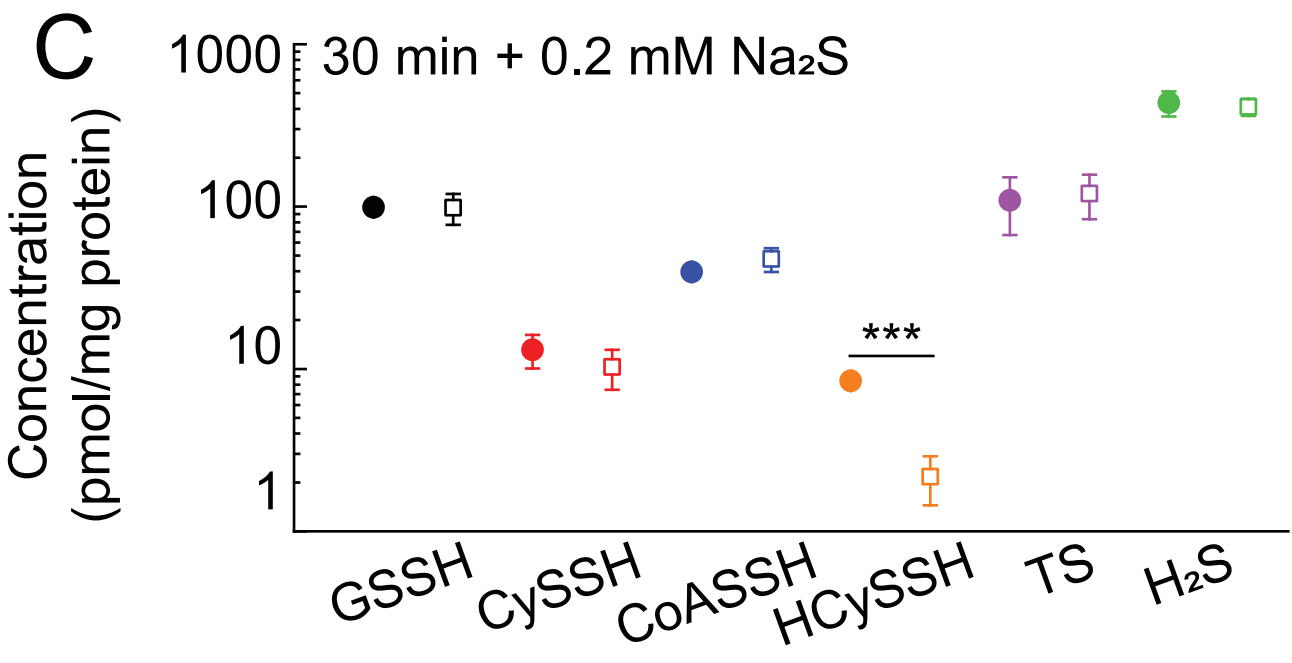

Supplement: FIG S1 [file mBio.01254-20-sf001.pdf]

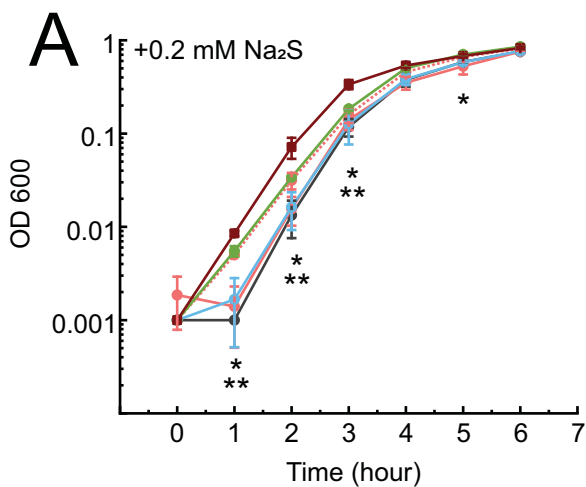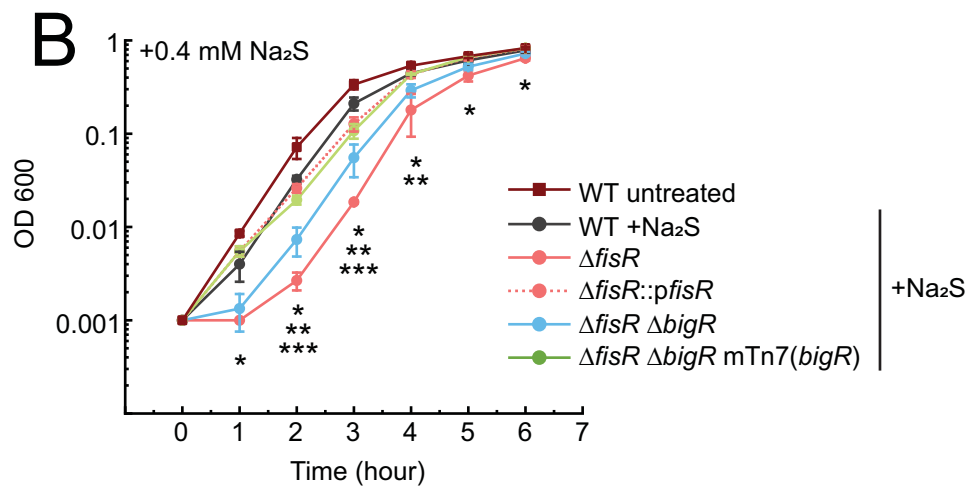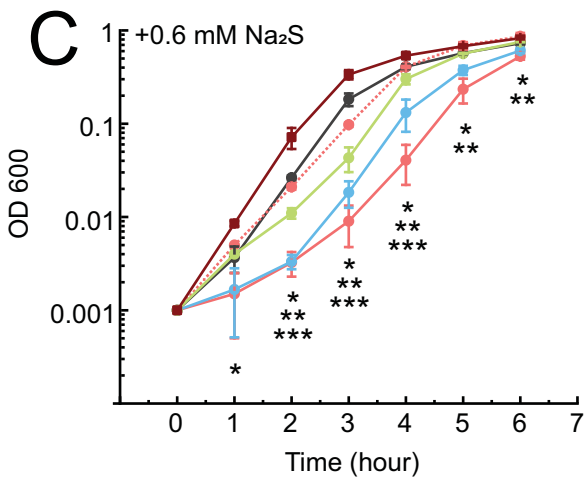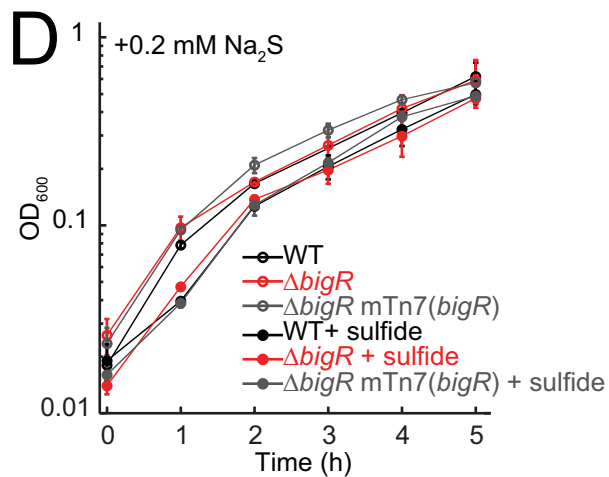

Supplement: FIG S2 [file mBio.01254-20-sf002.pdf]

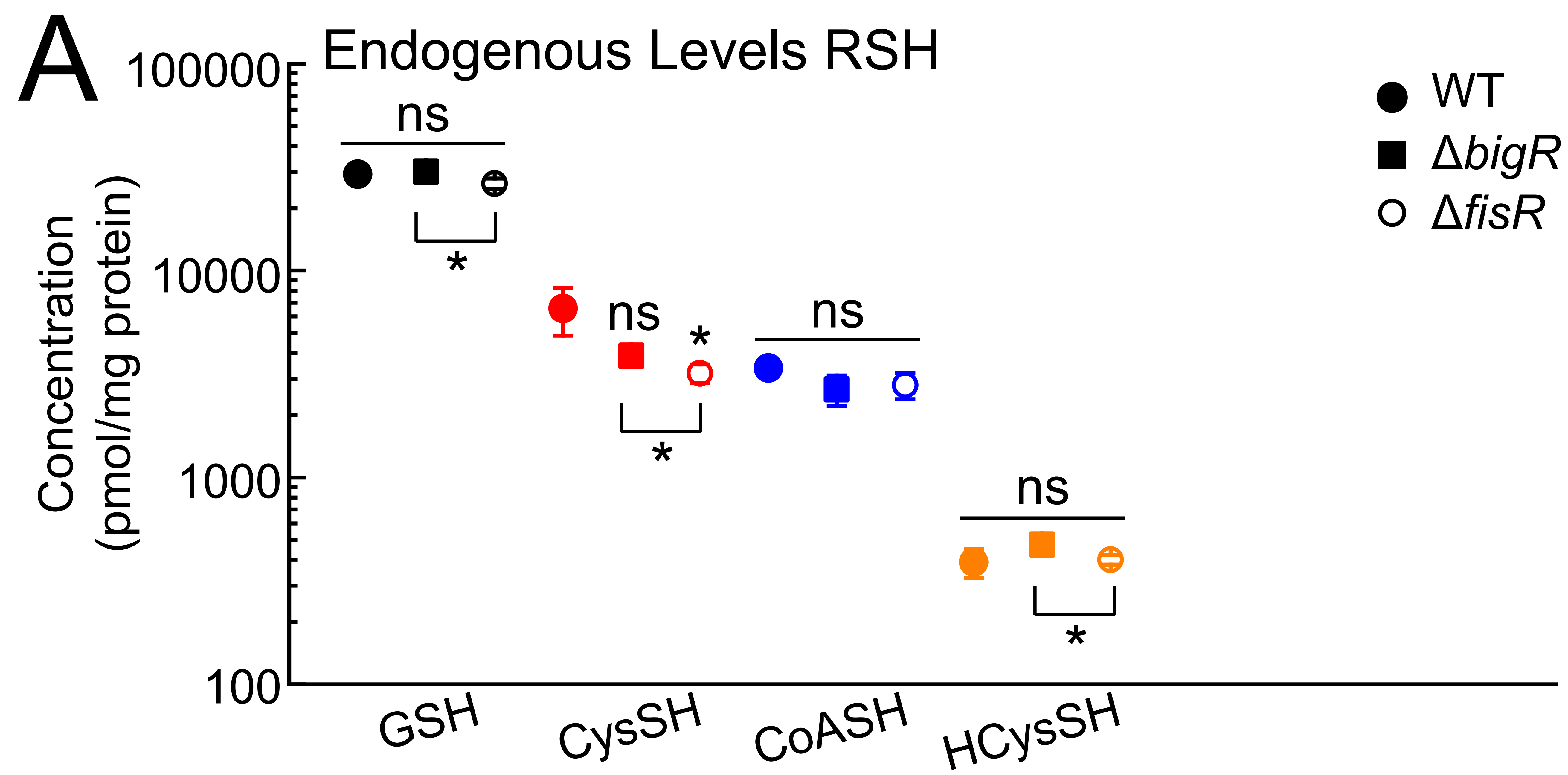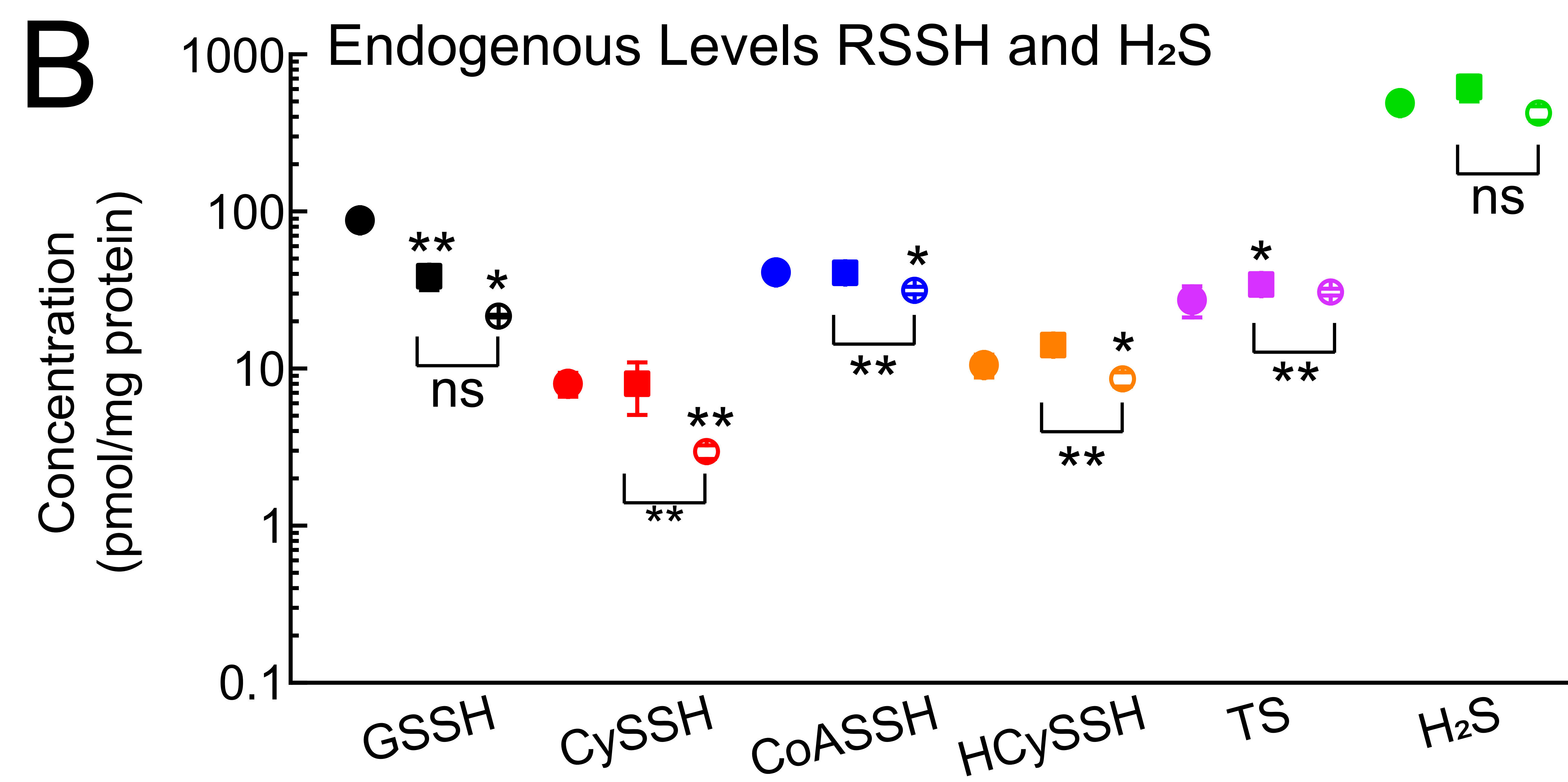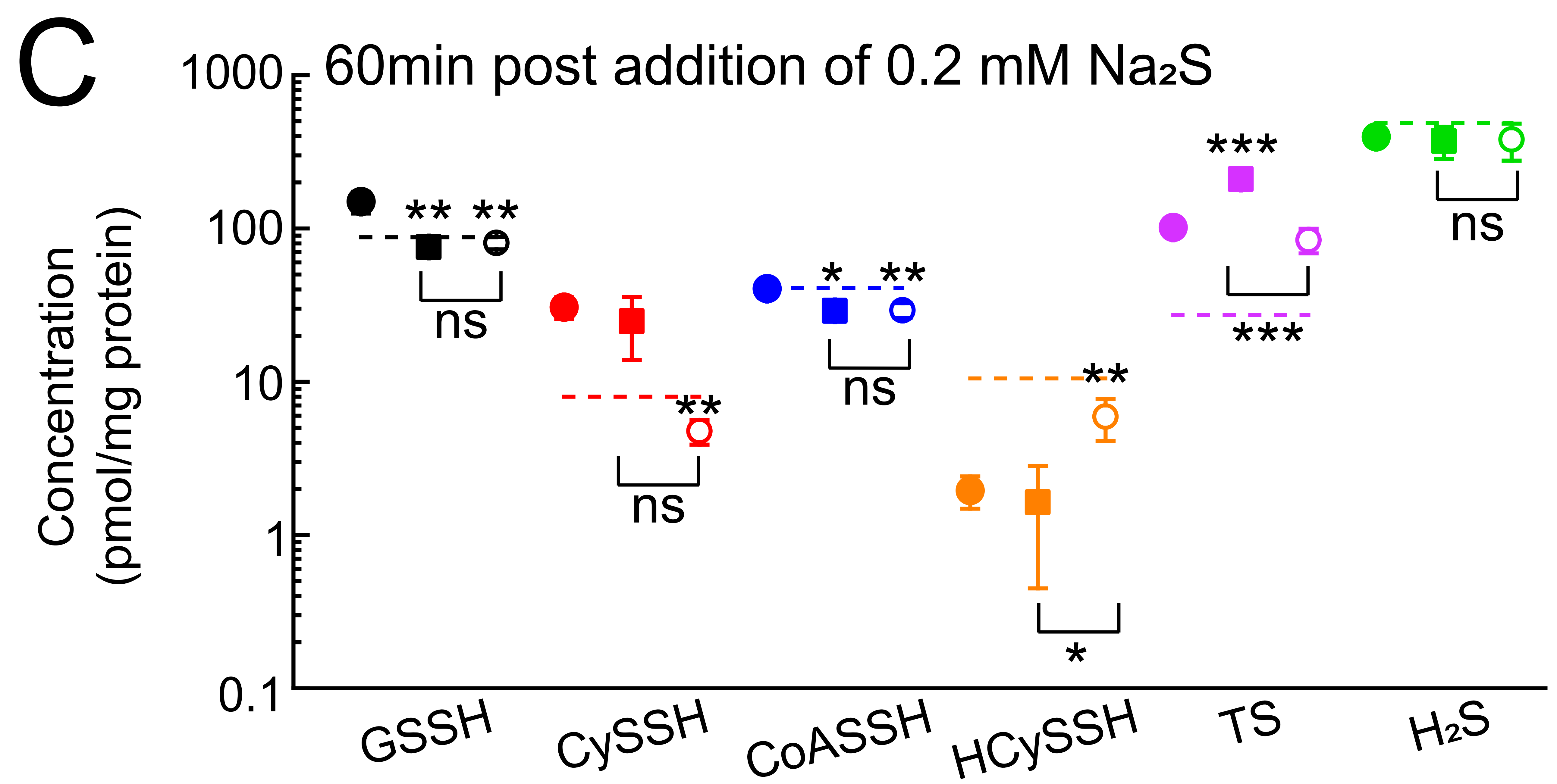

Supplement: FIG S3 [file mBio.01254-20-sf003.pdf]

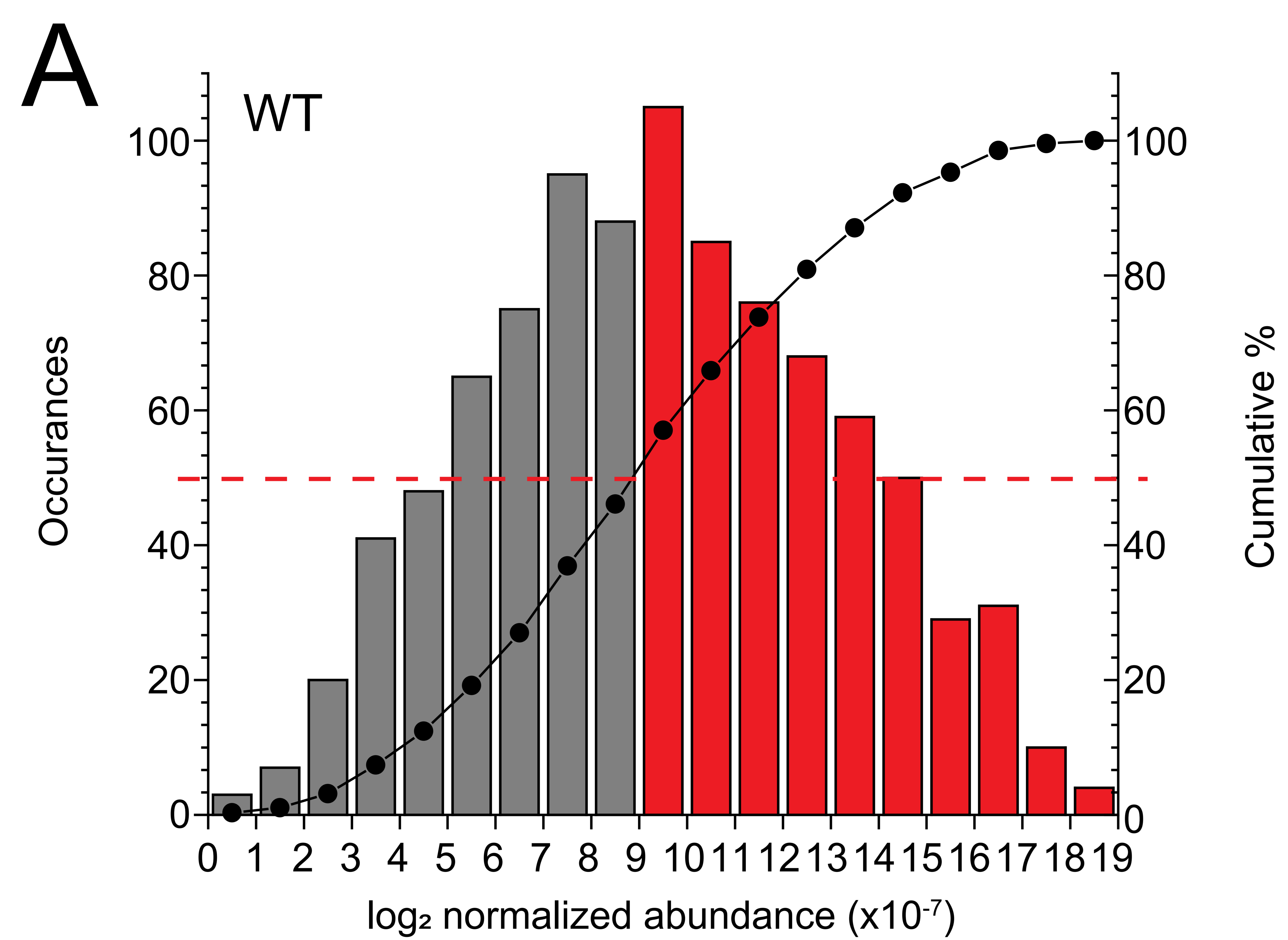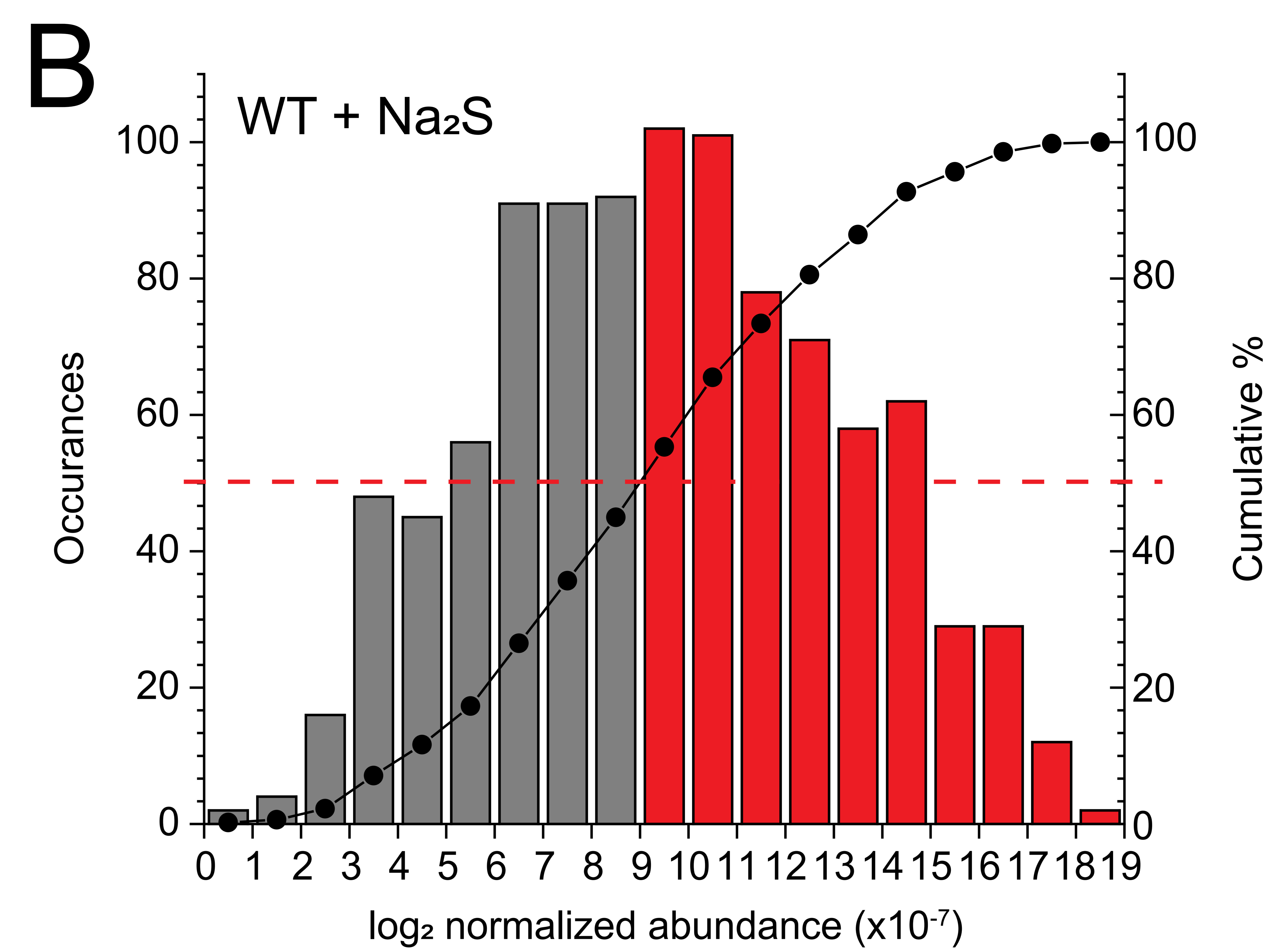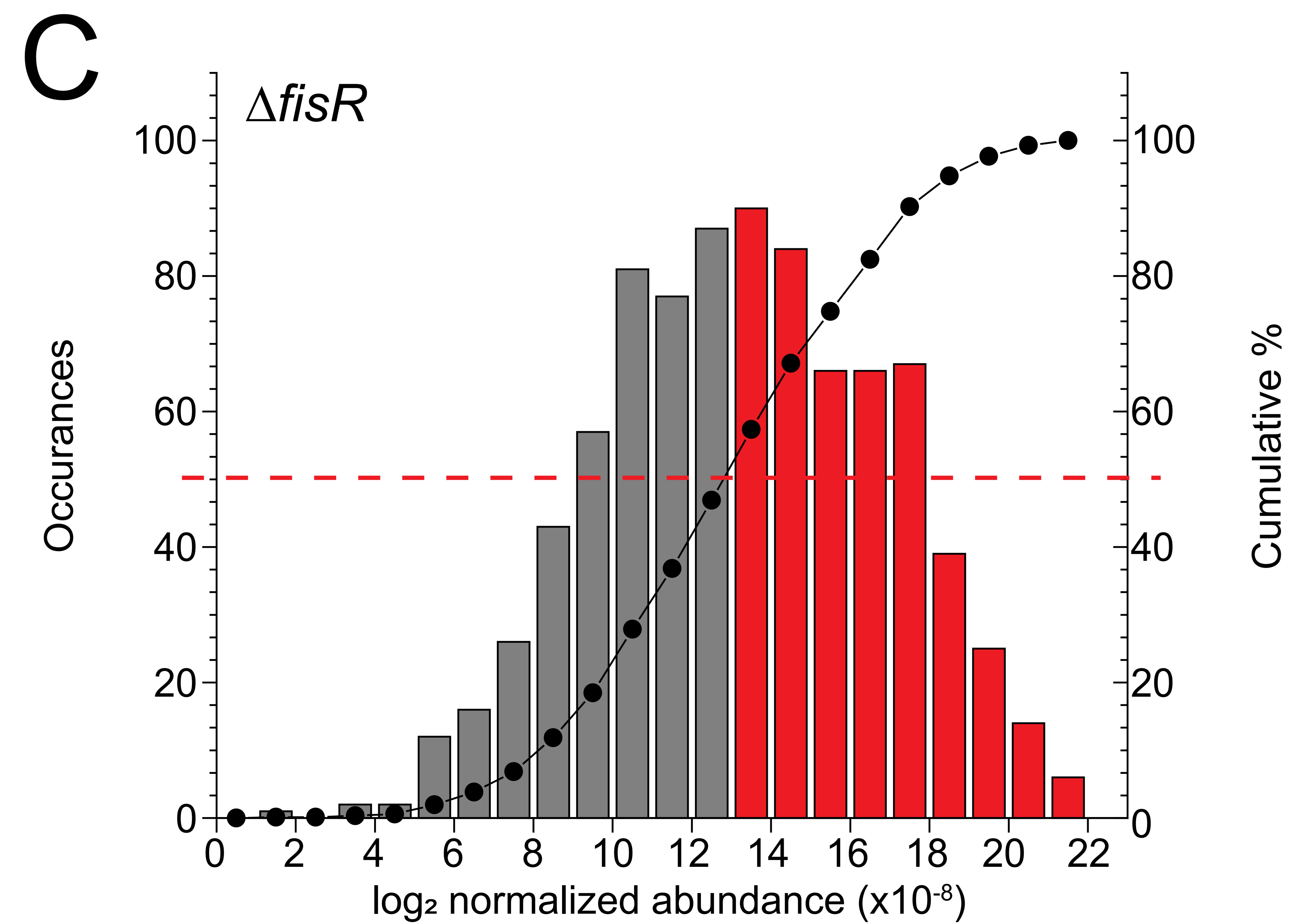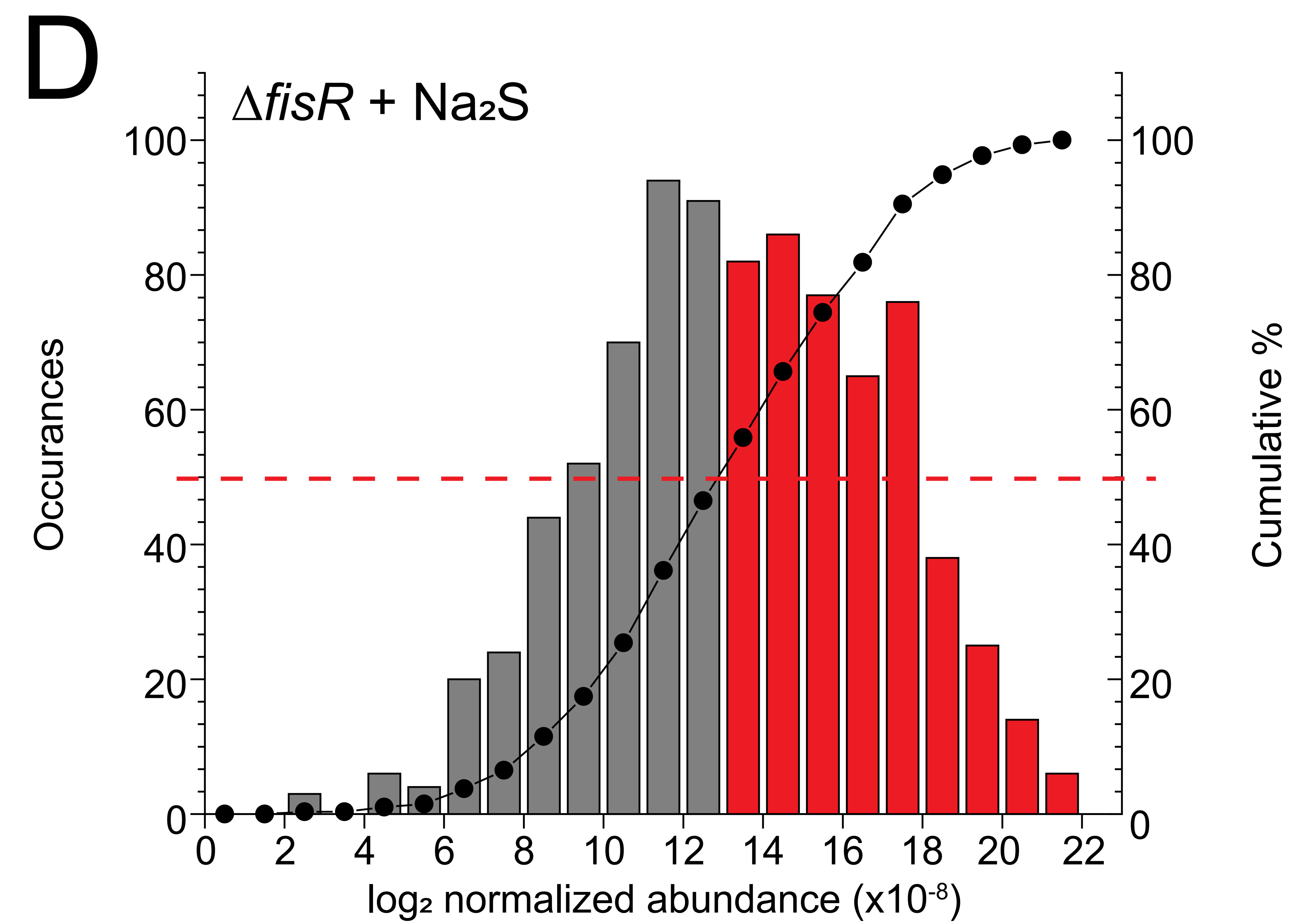

Supplement: FIG S4 [file mBio.01254-20-sf004.pdf]

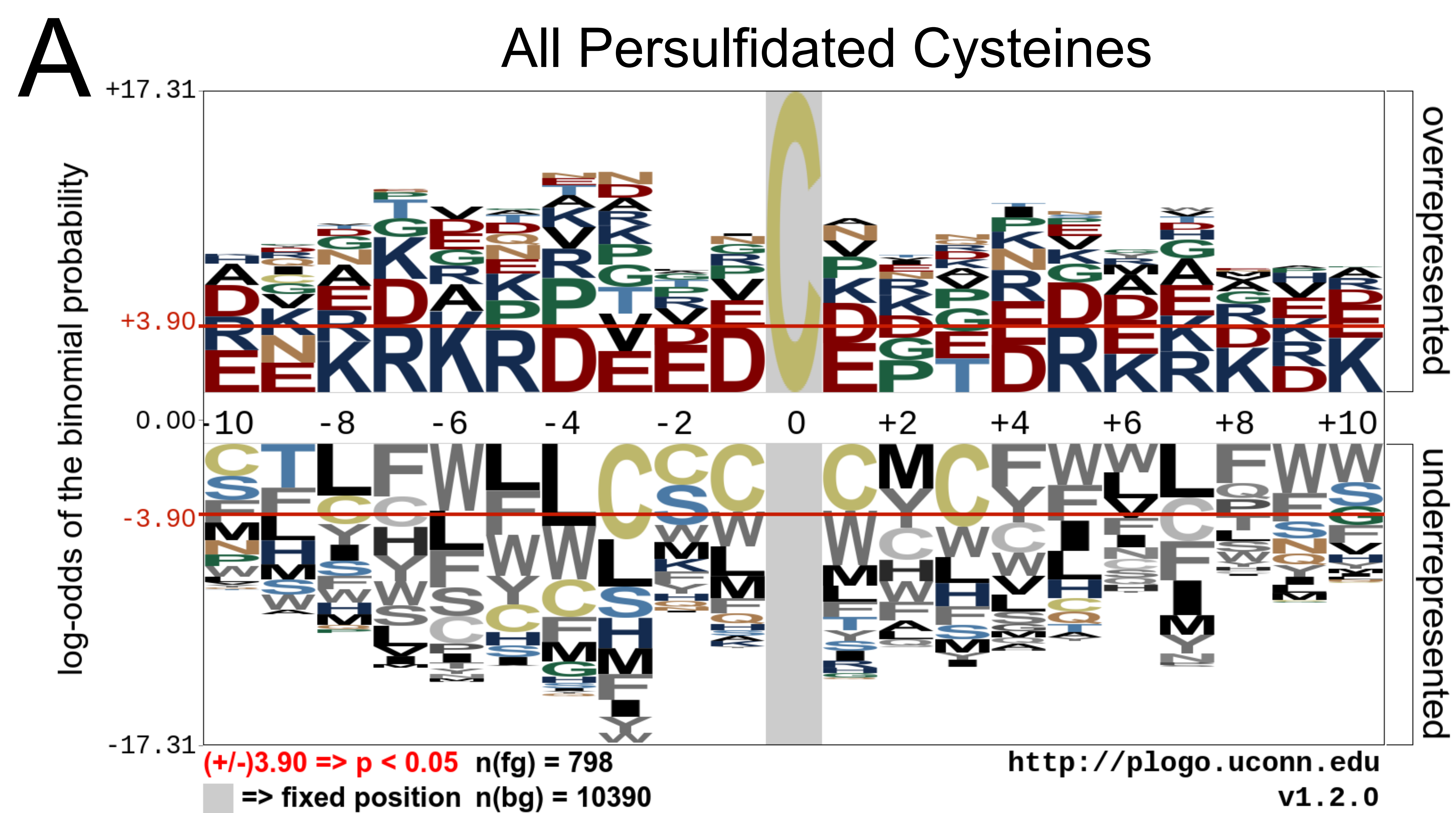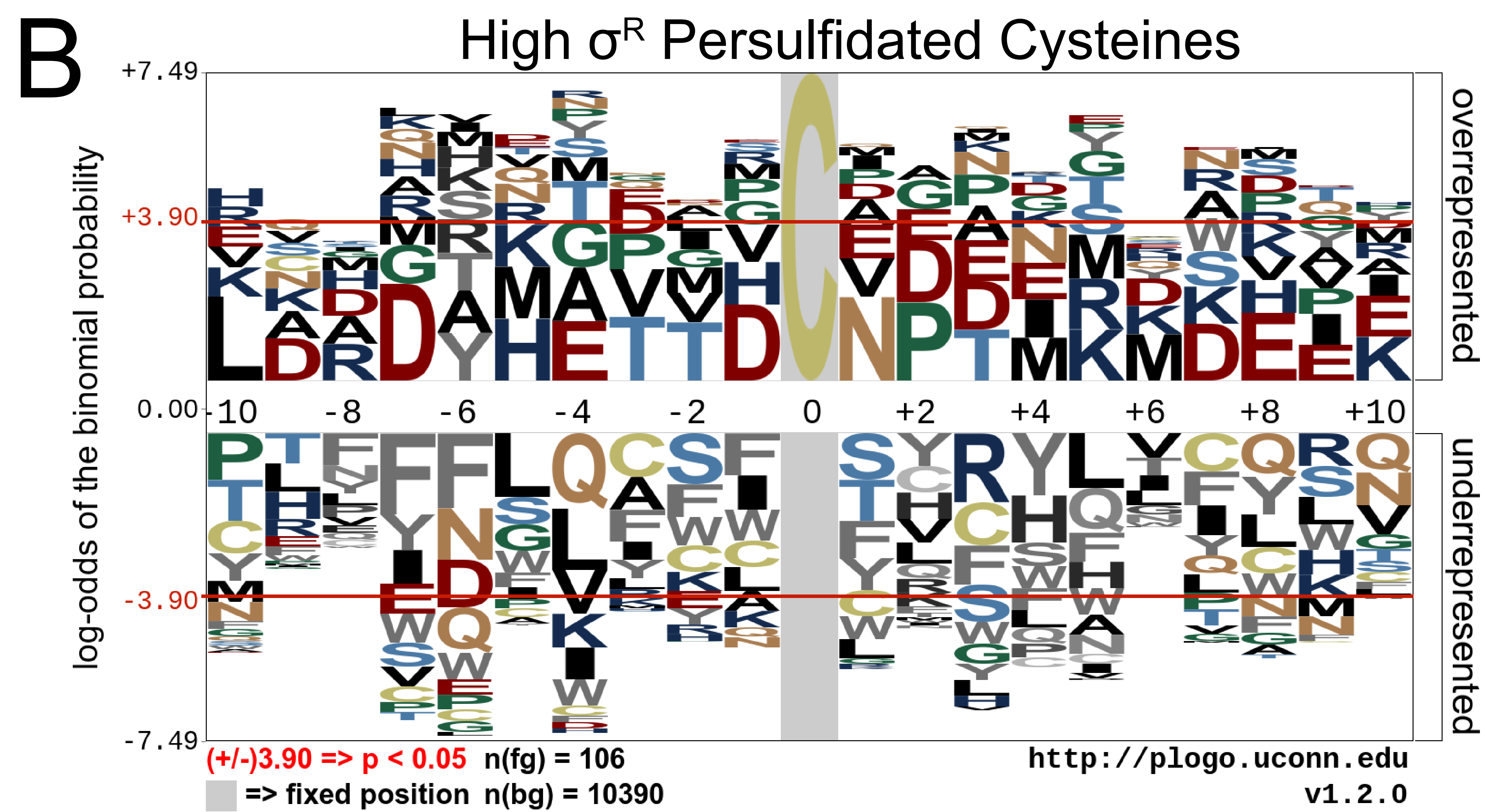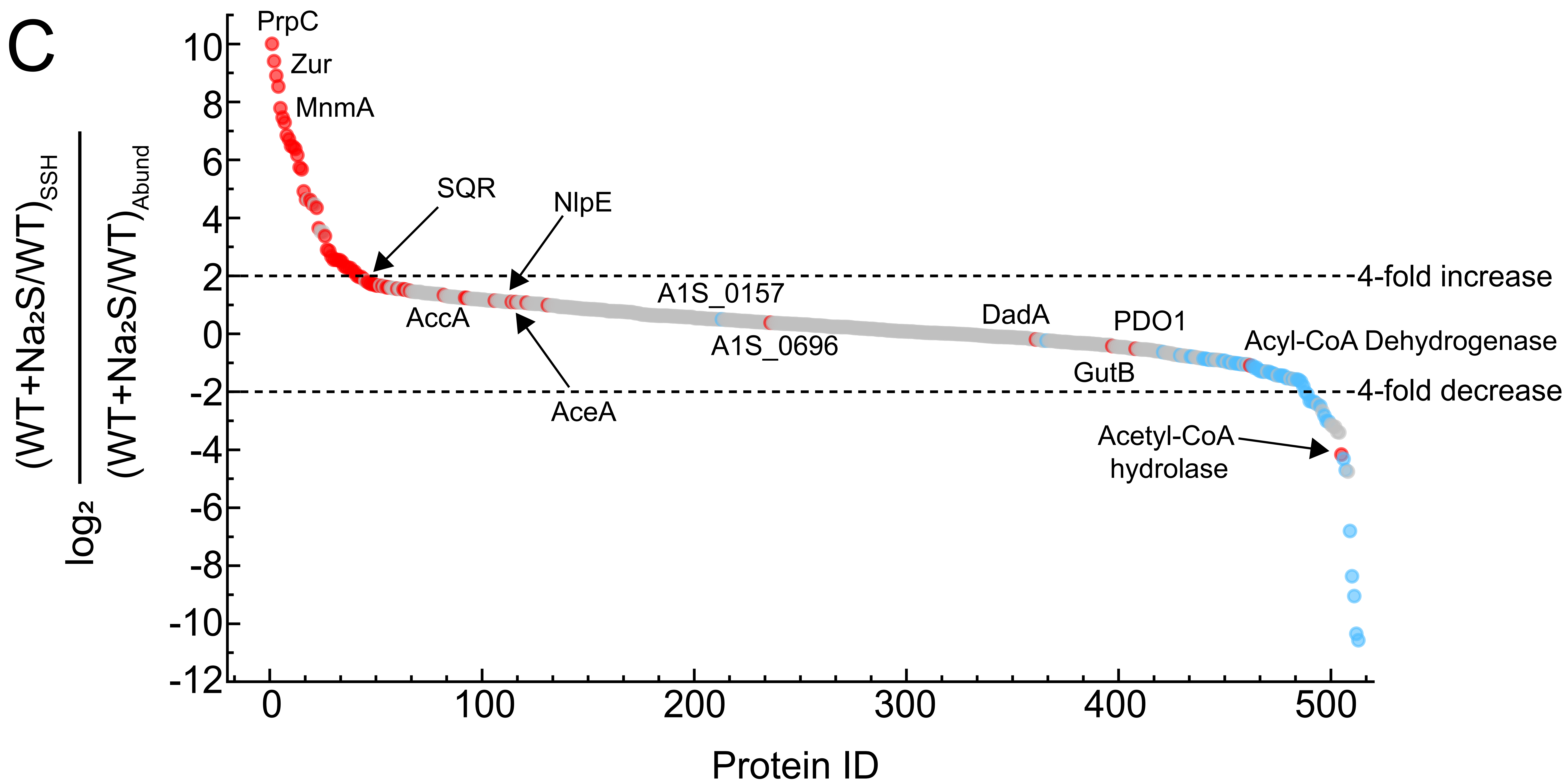

Supplement: FIG S7 [file mBio.01254-20-sf007.pdf]

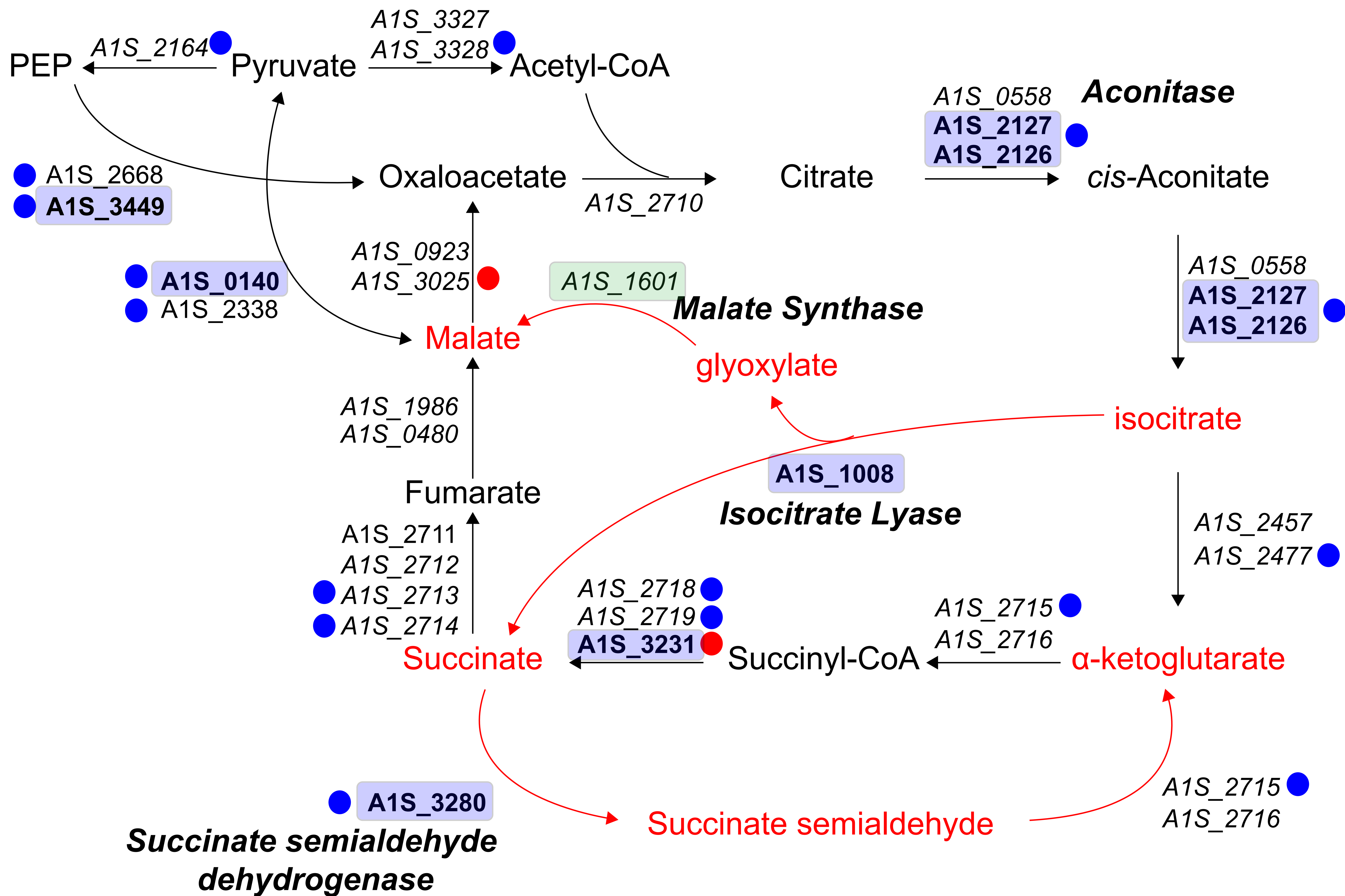

Supplement: FIG S8 [file mBio.01254-20-sf008.pdf]
