# Supplementary material for: The Response of Acinetobacter baumannii to Hydrogen Sulfide Reveals Two Independent Persulfide-Sensing Systems and a Connection to Biofilm Regulation
Source: mBio. 2020 Jun 23;11(3):e01254-20. doi: 10.1128/mBio.01254-20 (PMC7315123; doi:10.1128/mBio.01254-20)

Locus	Function	Cysteine (Y/N)	Persulfidated (Y/N)	Sigma ratio	Persulfidation Normalized
A1S_0140	nad-linked malate dehydrogenase	Y	Y	0.722	1.374
A1S_0412	catalase	Y	N	-	-
A1S_0800	bacterioferritin	Y	N	-	-
A1S_1008	isocitrate lyase	Y	Y	0.815	2.131
A1S_1708	PDO	Y	Y	1	0.701
A1S_1709	SQR	Y	Y	1	3.915
A1S_3175	bacterioferritin	Y	N	-	-
A1S_3280	succinate semialdehyde dehydrogenase	Y	Y	0.825	2.801
A1S_3449	phosphoenolpyruvate carboxylase	Y	Y	0.729	1.266

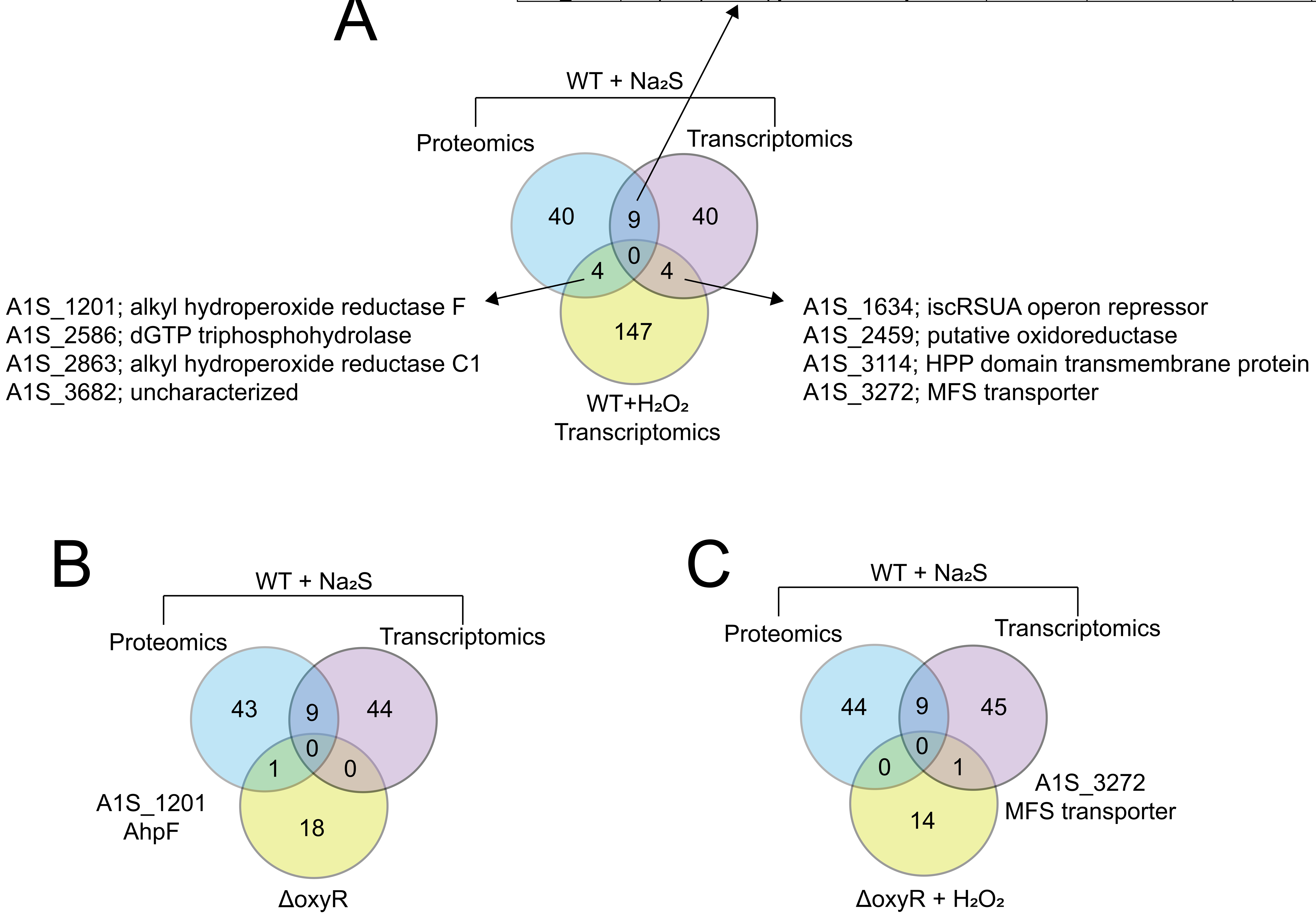

Supplement: FIG S5 [file mBio.01254-20-sf005.pdf]
