# Supplementary material for: The Response of Acinetobacter baumannii to Hydrogen Sulfide Reveals Two Independent Persulfide-Sensing Systems and a Connection to Biofilm Regulation
Source: mBio. 2020 Jun 23;11(3):e01254-20. doi: 10.1128/mBio.01254-20 (PMC7315123; doi:10.1128/mBio.01254-20)
Supplement: FIG S6 [file mBio.01254-20-sf006.pdf]

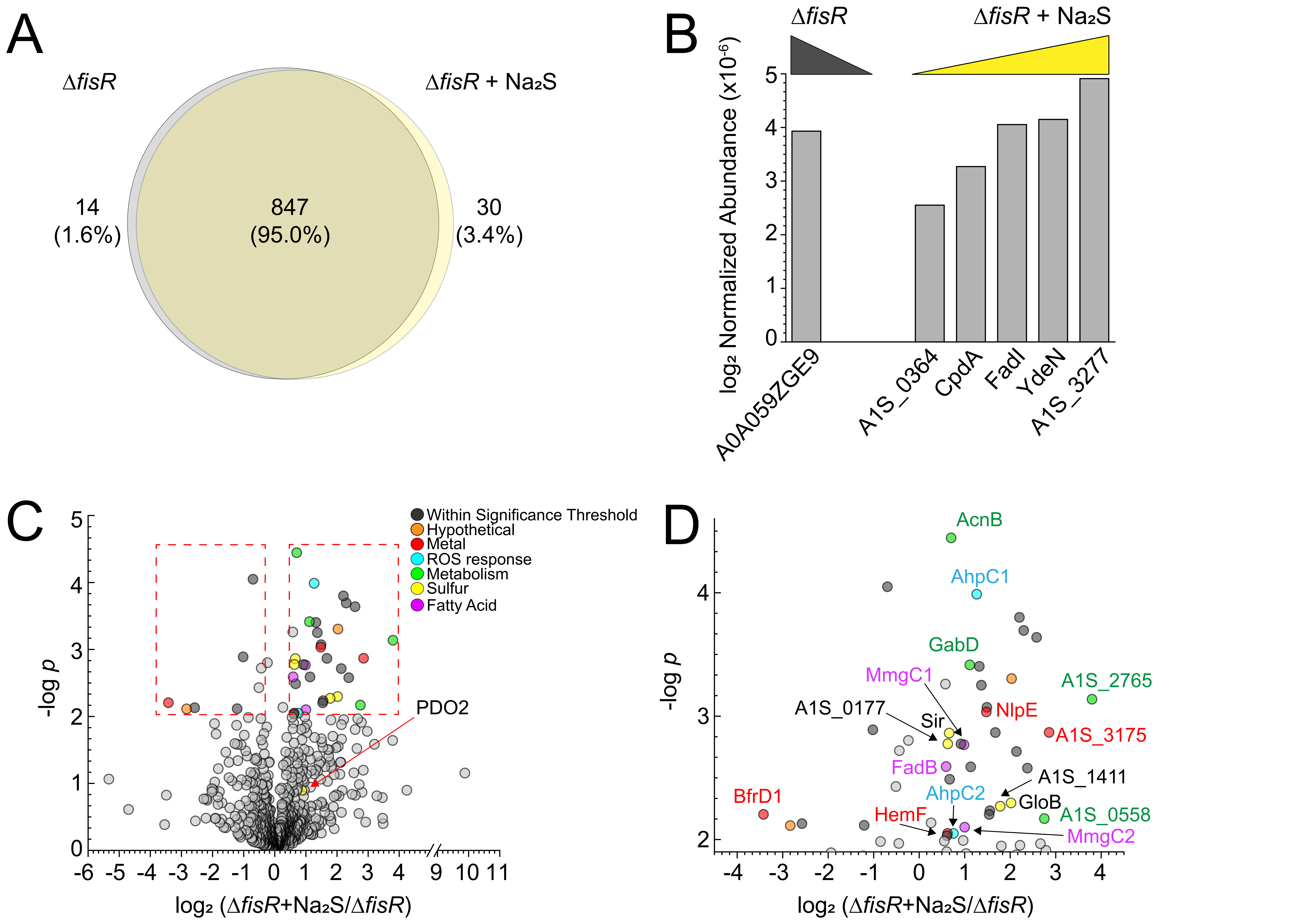
A
ΔfisR
ΔfisR + Na₂S
14
(1.6%)
847
(95.0%)
30
(3.4%)
B
ΔfisR
ΔfisR + Na₂S
log₂ Normalized Abundance (x10⁻⁶)
A0A059ZGE9
A1S_0364
CpdA
FadI
YdeN
A1S_3277
C
Within Significance Threshold
Hypothetical
Metal
ROS response
Metabolism
Sulfur
Fatty Acid
PDO2
-log p
log₂ (ΔfisR+Na₂S/ΔfisR)
D
AcnB
AhpC1
GabD
MmgC1
A1S_2765
A1S_0177
Sir
NlpE
A1S_3175
FadB
A1S_1411
BfrD1
AhpC2
GloB
A1S_0558
HemF
MmgC2
-log p
log₂ (ΔfisR+Na₂S/ΔfisR)
